# The neuropeptide FLP-11 induces and self-inhibits sleep through the receptor DMSR-1 in *Caenorhabditis elegans*

**DOI:** 10.1016/j.cub.2025.03.039

**Published:** 2025-04-23

**Authors:** Lorenzo Rossi, Kenneth Amoako, Inka Busack, Luca Golinelli, Amy Courtney, Judith Besseling, William Schafer, Isabel Beets, Henrik Bringmann

**Affiliations:** 1 Biotechnology Center, Center for Molecular and Cellular Bioengineering, Technische Universität Dresden, Am Tatzberg 47/49, 01307 Dresden, Germany; 2 Department of Biology, KU Leuven, Naamsestraat 59, 3000 Leuven, Belgium; 3 MRC Laboratory of Molecular Biology, Francis Crick Avenue, Cambridge Biomedical Campus, Cambridge CB2 0QH, UK; 4 Max Planck Institute of Biophysical Chemistry, Am Fassberg 11, 37077 Göttingen, Germany; 5 Faculty of Medicine Carl Gustav Carus, Technische Universität Dresden, Fetscherstraße 74, 01307 Dresden, Germany

## Abstract

Sleep is caused by the depolarization of sleep-active neurons, which secrete gamma-aminobutyric acid (GABA) and neuropeptides such as conserved RFamide (c-terminal Arg-Phe-NH_2_ motif) neuropeptides to dictate when an organism falls asleep and when it wakes up.^1–10^ However, the mechanisms by which neurotransmission from sleep-active neurons induces sleep and determines the duration of sleep remain poorly understood. Sleep in *Caenorhabditis elegans* crucially requires the single sleep-active RIS neuron, which induces sleep via the release of FLP-11 RFamide neuropeptides.^8,11^ However, how RIS and FLP-11 control sleep is not well understood, as the receptor through which FLP-11 acts has yet to be identified. In this study, we discovered that RIS and FLP-11 control sleep through the G_i/o_-protein coupled receptor DroMyoSuppressin receptor related 1 (DMSR-1).^12,13^ Using cell-specific knockdowns,^14^ we demonstrate that *dmsr-1* induces sleep by acting in cholinergic neurons downstream of RIS activation. Pharmacological intervention indicates that inhibiting cholinergic signaling is necessary for sleep. Consistently, DMSR-1 expression in cholinergic neurons is essential for core sleep functions, including protective gene expression and survival. In contrast, we found that *dmsr-1* in RIS mediates negative feedback control during sleep that limits RIS calcium activation and the duration of sleep. Consequently, *dmsr-1* in RIS inhibits protective gene expression and survival. Thus, DMSR-1 controls both the initiation and limitation of sleep, effectively coupling sleep induction with a sleep-stop signal. RFamide neuropeptide-GPCR signaling might underlie similar dual mechanisms of sleep control in other species, and self-inhibition of sleep-active neurons might represent a conserved mechanism for limiting the duration of sleep.

## Graphical abstract

**Graphical abstract:**
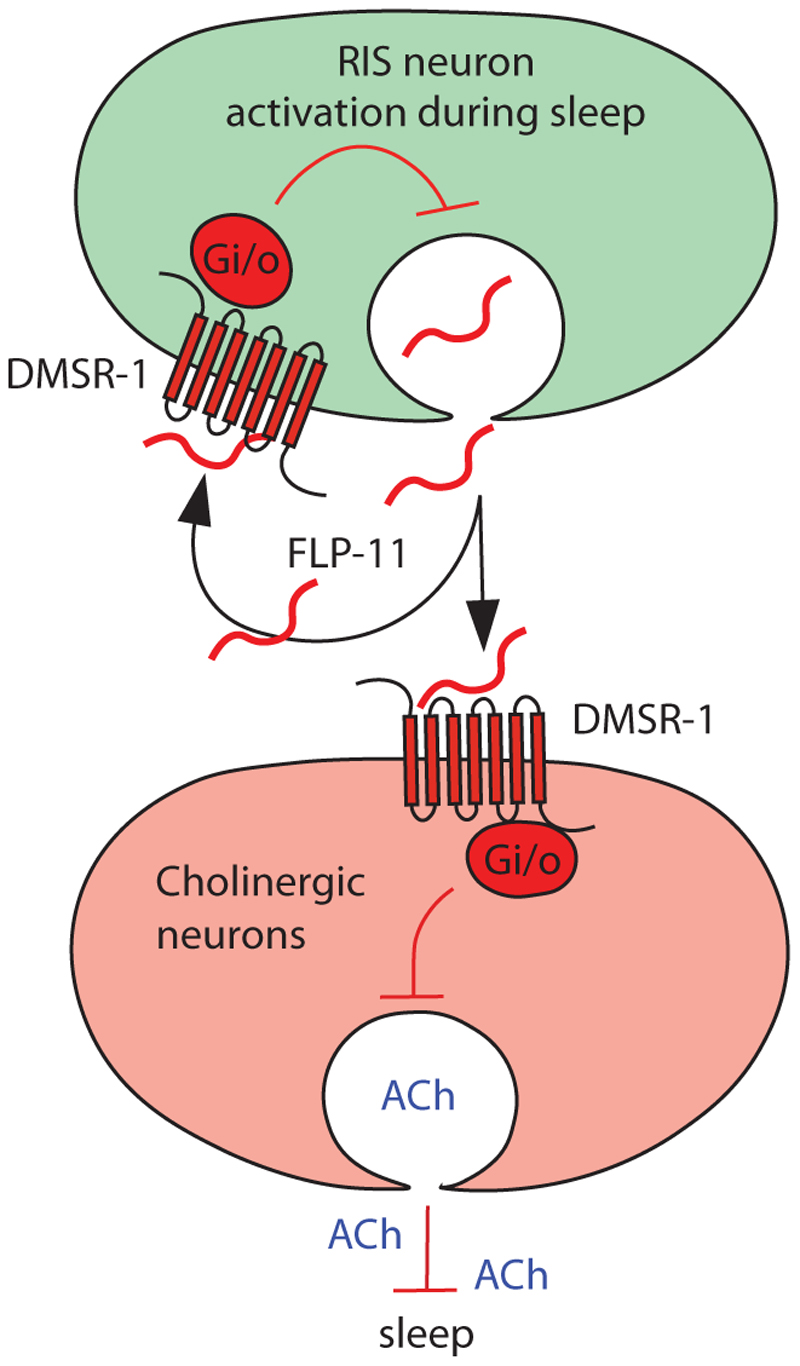

## Results

### The G-protein-coupled neuropeptide receptor DMSR-1 is required for sleep during L1 arrest

Recent large-scale *in vitro* screening identified potential receptors for FLP-11. Among these, DMSR-1 was the only receptor activated by FLP-11 with a half-maximal effective concentration (EC_50_) in the nanomolar to sub-nanomolar range, whereas FRPR-8 and DMSR-7 were activated with much lower potency.^13^ FRPR-8 and DMSR-7 have not yet been studied in relation to sleep. DMSR-1 was first identified as a receptor for FLP-13 neuropeptides,^12^ which are released from the ALA neuron to promote sleep upon cellular stress.^4,9^

We tested whether DMSR-1, FRPR-8, and DMSR-7 are required for sleep induction downstream of FLP-11. To avoid any interference from FLP-13 in our sleep measurements, we conducted our study in the absence of overt stressors known to activate ALA. When *C. elegans* larvae hatch without food, they arrest development at the first (L1) larval stage^15,16^ and increase sleep via RIS^17^ and FLP-11.^18^ This L1 arrest sleep in the absence of additional cellular stress is not known to be regulated by ALA^19^ or FLP-13, providing an ideal experimental model to study the regulation of sleep via FLP-11 receptors, including DMSR-1.

**Figure 1:**
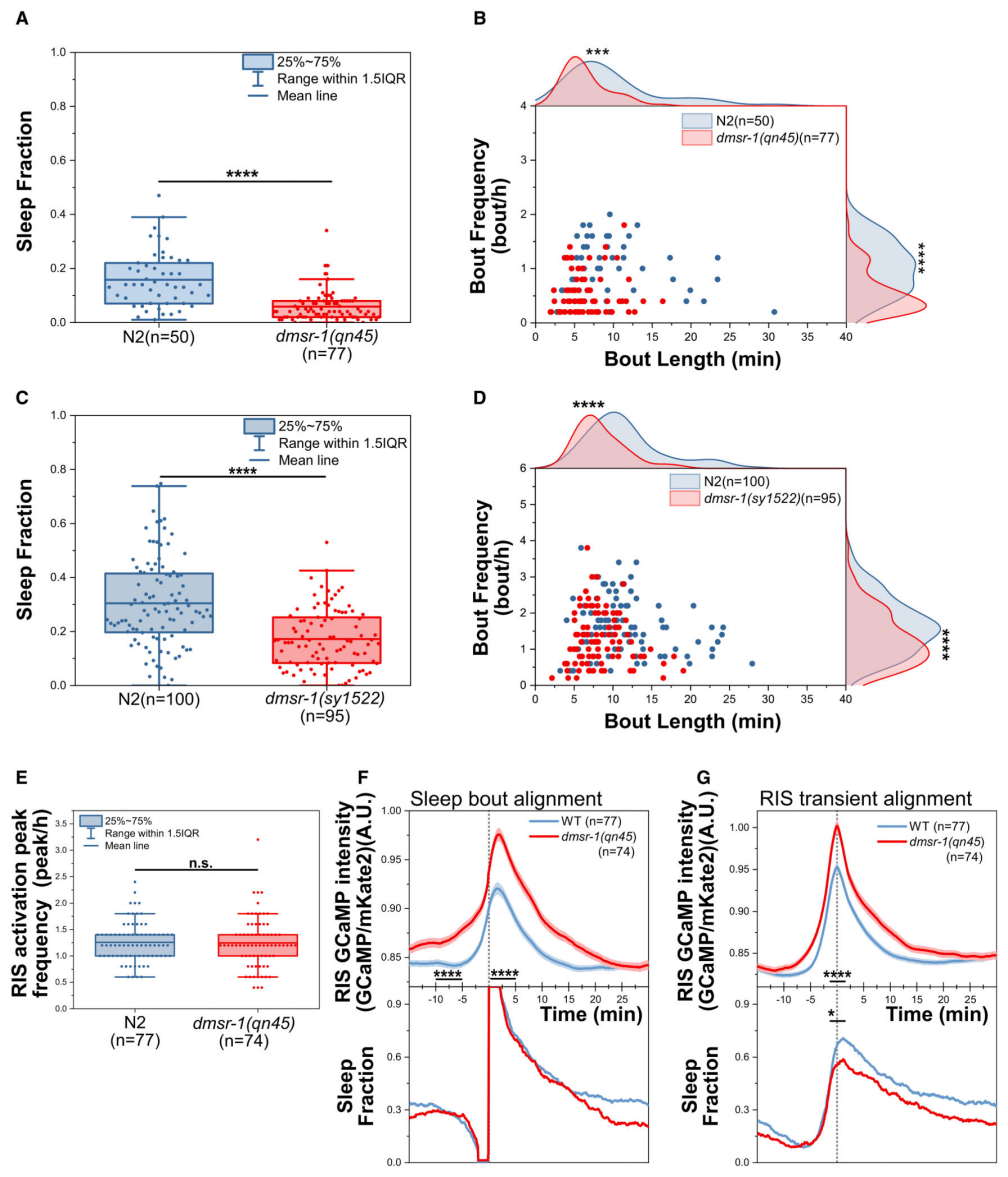
The G-protein-coupled neuropeptide receptor DMSR-1 is required for sleep during L1 arrest (A and B) *dmsr-1(qn45)* reduces (A) the fraction of time spent sleeping as well as (B) sleep bout frequency and length. Distribution curves were calculated using kernel smoothing. N2: *n* = 50, *dmsr-1(qn45)*: *n* = 77 animals, with 3 biological replicates. ***p ≤ % 0.001, ****p ≤ % 0.0001, Wilcoxon rank-sum test. (C and D) *dmsr-1(sy1522)* reduces (C) the fraction of time spent sleeping as well as (D) sleep bout frequency and length. Distribution curves were calculated using kernel smoothing. N2: *n* = 100, *dmsr-1(sy1522)*: *n* = 95 animals, with 3 biological replicates. ****p ≤ % 0.0001, Wilcoxon rank-sum test. (E–G) RIS calcium activity is elevated in *dmsr-1(qn45). N2*: *n* = 77, *dmsr-1(qn45)*: *n* = 74 animals, with 3 biological replicates, animals were imaged for 5 h. *(E) dmsr-1(qn45)* does not affect the frequency of RIS activation. We extracted all RIS activation transients during the 5-h recording, regardless of whether they were associated with sleep bouts. n.s. = not significant (*p* > 0.05), Wilcoxon rank-sum test. (F) Sleep bout onset alignment shows elevated RIS activity in *dmsr-1(qn45)* before (10–5 min before sleep bout onset) and during RIS activation (the 5 min following sleep onset). ****p ≤ % 0.0001, Wilcoxon rank-sum test. (G) Alignment of RIS calcium activation transients to their peak shows that *dmsr-1(qn45)* has stronger RIS activity but a lower probability of entering sleep during RIS activation. n.s. = not significant (*p* > 0.05), *p ≤ % 0.05, ****p ≤ % 0.0001, Wilcoxon rank-sum test (−1 to 1 min relative to the peak). See also the related Figure S1 and Data S1 and S4.

We cultured mutants of *dmsr-1*,^12,20^ *frpr-8*, and *dmsr-7* in agarose hydrogel microfluidic chambers and imaged their sleep behavior during L1 arrest.^21,22^ We calculated the fraction of time spent sleeping, and recorded the length and frequency of sleep bouts.^17,18^ In the *dmsr-1(qn45)*^12^ and *dmsr-1(sy1522)*^20^ mutants, the fraction of time spent sleeping decreased by about half, with both the length and frequency of sleep bouts reduced (Figures 1A–1D). The other receptor mutants did not exhibit any detectable sleep phenotypes, and we did not investigate them further (Figure S1).

Next, we used GCaMP to monitor calcium activity in RIS across the sleep-wake cycle in *dmsr-1(qn45)*. To average the data, we aligned all sleep bouts to their onset^17,23^ and also aligned RIS calcium transients to their maximum. The frequency of RIS activation transients in *dmsr-1(qn45)* mutant larvae was similar to that in the wild type (Figure 1E). When we aligned sleep bouts, we found that both the overall calcium baseline activity and the maximum activation of RIS during sleep were higher in *dmsr-1* mutant larvae (Figure 1F). RIS calcium activation peak alignment showed a significantly increased peak maximum, which, however, was associated with a reduced proportion of animals sleeping (Figure 1G). Therefore, DMSR-1 is required for sleep during L1 arrest. It appears to play a dual role in sleep regulation, being necessary for sleep induction while also exerting an inhibitory effect on RIS.

### DMSR-1 is required for sleep downstream of RIS and FLP-11

DMSR-1 is a promiscuous receptor, with FLP-11, FLP-13, and FLP-25 being the most potent ligands.^13^ Although *flp-11* is known to regulate sleep during L1 arrest,^18^ it is not known whether *flp-13* and *flp-25* control sleep during this stage. We hence measured the sleep duration in mutants of these neuropeptide genes.^4,8,9,24^ Overall sleep duration in *flp-13* mutants was not altered, while *flp-25* mutants exhibited a slight increase in sleep duration. In contrast, *flp-11* mutation reduced the time spent sleeping by more than half due to decreases in both the frequency and length of sleep bouts (Figures S2A–S2D). Thus, FLP-11 neuropeptides are the primary activators of DMSR-1 required for sleep during L1 arrest.

**Figure 2:**
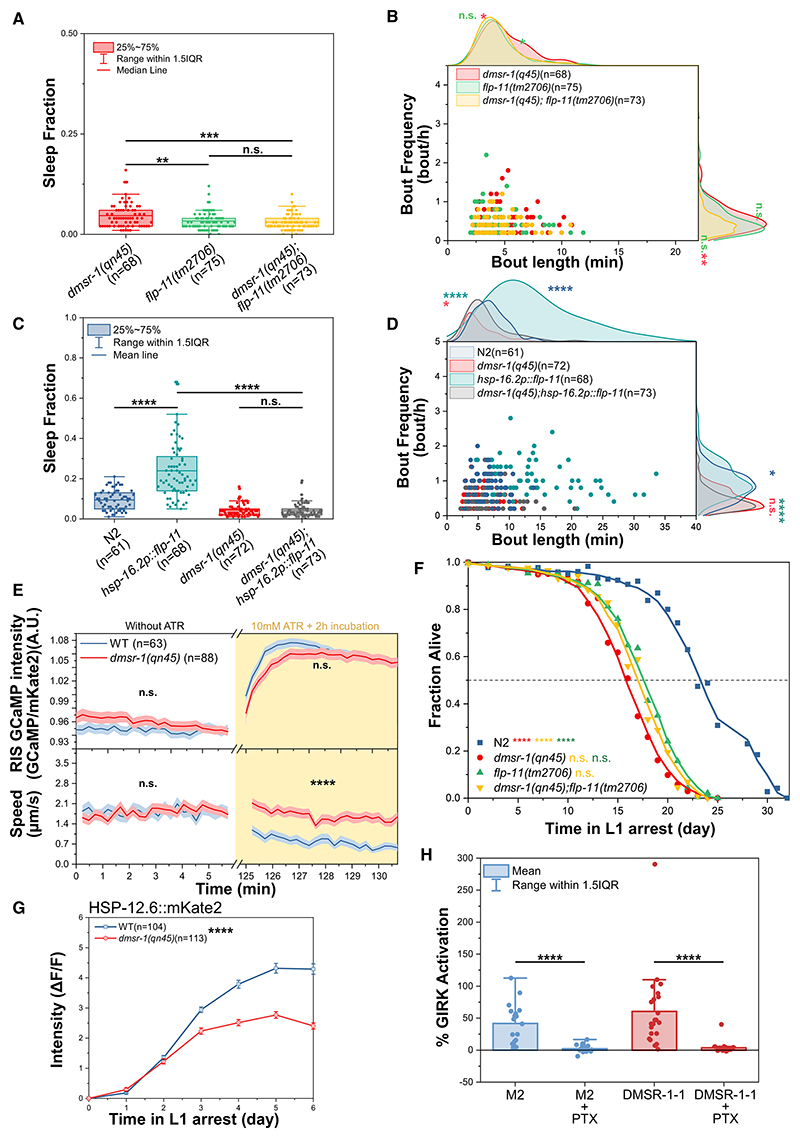
DMSR-1 is coupled to G_i/o_ and is required for sleep downstream of RIS-released FLP-11 (A and B) *flp-11(tm2706)* causes more severe sleep loss than *dmsr-1(qn45)*, and the *dmsr-1(qn45); flp-11(tm2706)* double mutant resembles the *flp-11(tm2706)* single mutant. Bout frequency and length are reduced in the double mutant compared with *dmsr-1(qn45)* but are not significantly different from those in the *flp-11(tm2706)* single mutant. *dmsr-1(qn45)*: *n* = 68, *flp-11(tm2706)*: *n* = 74, *dmsr-1(qn45); flp-11(tm2706)*: *n* = 73, with 3 biological replicates. n.s. = not significant (*p* > 0.05), *p ≤ % 0.05, **p ≤ % 0.01, ***p ≤% 0.001, Wilcoxon rank-sum test. (C and D) *flp-11* overexpression leads to nearly a 3-fold increase in sleep, which is suppressed by *dmsr-1(qn45)*. N2: *n* = 61, *hsp-16*.*2p::flp-11*: *n* = 68, *dmsr-1(qn45); hsp-16*.*2p::flp-11*: *n* = 73, with 3 biological replicates. n.s. = not significant (*p* > 0.05), *p ≤ % 0.05, *p ≤ % 0.05, ****p ≤ % 0.0001, Wilcoxon rank-sum test. (E) Optogenetic stimulation of RIS was performed using *flp-11p::ReaChR::flp-11-3’UTR* during L1 arrest. We first recorded behavior and RIS calcium activity under baseline conditions. Because calcium imaging illumination can activate ReaChR during baseline imaging when retinal is present, we measured the baseline in the absence of retinal. After baseline imaging, we added retinal and activated RIS with orange light while continuing to record the calcium sensor signal. Optogenetic activation of RIS increased RIS calcium concentration in both the wild-type background and in *dmsr-1(qn45)*. RIS activation reduces movement speed in wild-type worms but not in *dmsr-1(qn45)*. The Wilcoxon rank-sum test was performed for the time intervals from 0 to 5 min and from 125 to 130 min. Wild-type background: *n* = 63, *dmsr-1(qn45)*: *n* = 88, with 3 biological replicates. n.s. = not significant (*p* > 0.05), ****p ≤ % 0.0001, Wilcoxon rank-sum test. *(F) dmsr-1* is required for survival during L1 arrest. The *dmsr-1(qn45), flp-11(tm2706)*, and *dmsr-1(qn45); flp-11(tm2706)* double mutants reduce survival to a similar extent. We averaged survival data from three biological replicates. Approximately 100 animals were assayed per replicate for each genotype at every time point. Fisher’s exact test was conducted at the median survival of the mutants, which corresponds to day 16 for *dmsr-1(qn45)* and *dmsr-1(qn45); flp-11(tm2706)* and to day 18 for *flp-11(tm2706)*. n.s. = not significant (*p* > 0.05); ****p ≤ % 0.0001, Fisher’s exact test. *(G) dmsr-1* is required for the expression of *hsp-12*.*6*. ΔF/F was calculated as the change in fluorescence relative to the fluorescence level on day 0. In the wild-type background: *n* = 104, and for *dmsr-1(qn45)*: *n* = 113, with 3 biological replicates. The Wilcoxon rank-sum test was performed by averaging the fluorescence intensity per worm over the last 4 days. ****p ≤ % 0.0001. (H) DMSR-1 is coupled to G_i/o_. Activation of DMSR-1 by the FLP-11-3 peptide induces a strong current through a G-protein-coupled inward potassium channel, which is abolished by the G_i/o_ inhibitor pertussis toxin (PTX). The known G_i/o_-coupled receptor muscarinic M2 receptor was used as a positive control. M2 + Kir3.1/3.2: *n* = 18, M2 + Kir3.1/3.2 + PTX: *n* = 19, DMSR-1-1 + Kir3.1/3.2: *n* = 23, DMSR-1-1 + Kir3.1/3.2 + PTX: *n* = 23, 2 biological replicates. ****p ≤ % 0.0001, Wilcoxon rank-sum test. See also Figure S2 and Data S1 and S4.

Next, we tested whether *flp-11* and *dmsr-1* function in the same pathway to regulate sleep. First, we compared the sleep phenotypes of *flp-11(tm2706)* and *dmsr-1(qn45)* single mutants with that of the double mutant, which exhibited the same sleep phenotype as the *flp-11* single mutant (Figures 2A and 2B), suggesting that *dmsr-1* and *flp-11* act in the same pathway. We then tested whether increased sleep caused by overexpression of FLP-11^8^ depends on DMSR-1. We applied a mild heat stimulus to arrested L1 larvae to induce FLP-11 overexpression without triggering stress-induced sleep.^19,25^ The increased sleep caused by the overexpression of FLP-11 was completely suppressed in the absence of functional DMSR-1 (Figures 2C and 2D). These findings indicate that FLP-11 acts through DMSR-1 to induce sleep.

To test whether *dmsr-1* functions downstream of RIS, we optogenetically activated RIS in the *dmsr-1(qn45)* mutant. We used ReaChR to activate RIS, and simultaneously monitored its calcium activity using GCaMP.^23^ Optogenetic stimulation caused strong activation of RIS in both *wild-type* and *dmsr-1* mutant larvae. In *wild-type* larvae, the optogenetic stimulation strongly reduced mobility, whereas optogenetic immobilization was not detectable in *dmsr-1* mutant larvae (Figure 2E). These results indicate that *dmsr-1* is required for sleep downstream of RIS activation.

### *dmsr-1* is required for survival and protective gene expression

RIS releases FLP-11 during sleep to promote survival and protective gene expression during L1 arrest.^17,18,26^ We therefore investigated whether DMSR-1 is similarly necessary for survival and protective gene expression. First, we measured L1 arrest survival and recovery^15,17^ in *dmsr-1(qn45)* and *flp-11(tm2706)* single mutants as well as in the double mutant. All three mutants exhibited a similar decrease in both survival (Figure 2F) and recovery (Figure S2E). To assess protective gene expression in the *dmsr-1(qn45)* mutant, we measured the expression of the heat shock protein HSP-12.6, using a fluorescent mKate2 translational reporter as a marker for the transcriptional response to starvation.^26^ In *dmsr-1* mutant larvae, HSP-12.6::mKate2 expression was significantly reduced (Figure 2G). In summary, *dmsr-1* is critical for key sleep functions, including survival and protective gene expression during L1 arrest.

### DMSR-1 is coupled to G_i/o_

Previous genetic studies showed that G_i/o_ genes act downstream of *dmsr-1*, suggesting that DMSR-1 could be a G_i/o_-coupled receptor.^12^ To identify which G protein alpha subunit is associated with DMSR-1, we expressed the receptor in *Xenopus laevis* oocytes and used a two-electrode voltage clamp (TEVC) setup to measure receptor activation in response to FLP-11-3, the most potent of the four FLP-11 peptides.^13^ DMSR-1 exhibited a strong response to FLP-11-3, resulting in a significant increase in K^+^ current through G protein inward rectifying potassium (GIRK) channels. This response was abolished by pertussis toxin (PTX), which functionally uncouples the G_i/o_ protein subunit from their associated GPCRs^27^ (Figure 2H). As a control, we used the muscarinic M2 receptor, which is known to be G_i/o_ coupled and activated it by perfusion with its agonist, acetylcholine.^27^ Treatment with PTX inhibited the activation of the M2 receptor to the same extent as it did for DMSR-1 (Figure 2H). Altogether, these results demonstrate that DMSR-1 is a G_i/o_-coupled receptor.

### Cell-specific knockdown studies show that *dmsr-1* in cholinergic neurons is essential for sleep, whereas *dmsr-1* in GABAergic neurons inhibits sleep

**Figure 3:**
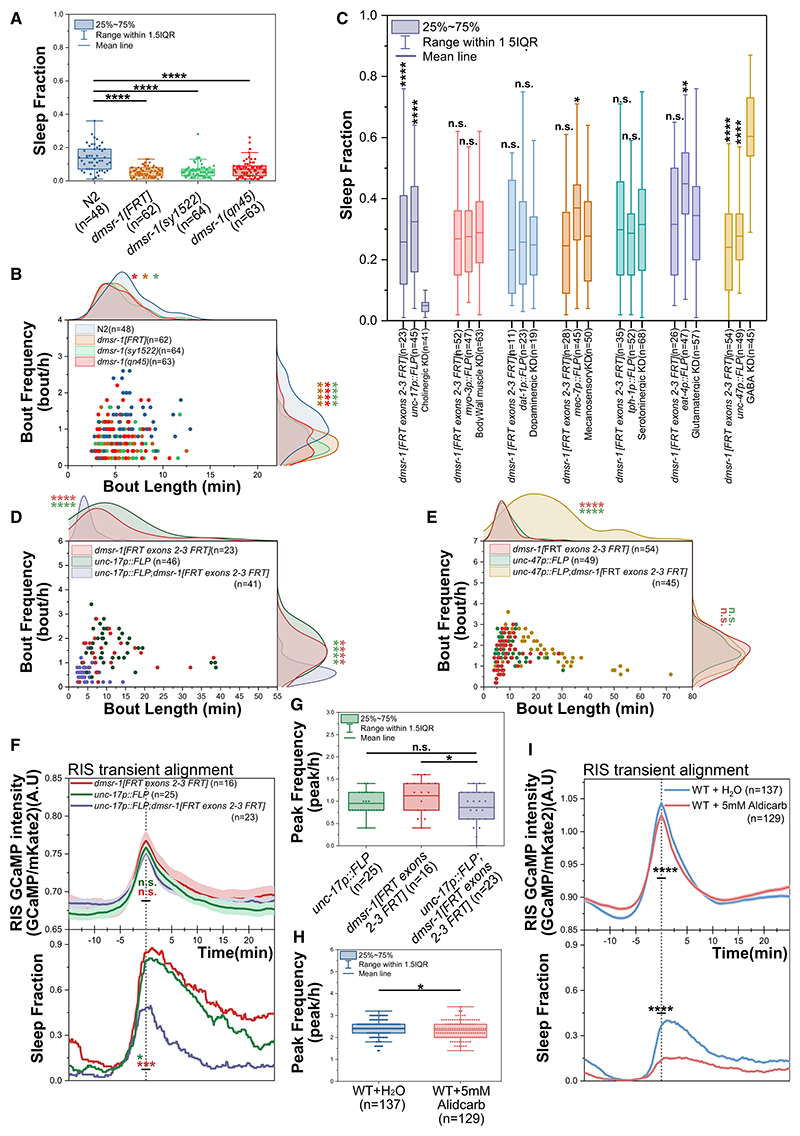
DMSR-1 acts in cholinergic neurons to promote sleep during RIS activation (A and B) To assess whether recombination of *dmsr-1[FRT exons 2-3 FRT]* results in a loss-of-function phenotype similar to *dmsr-1(qn45)* and *dmsr-1(sy1522)*, we generated a constitutive *dmsr-1* deletion via germline recombination, which we named *dmsr-1[FRT]*. Recombination between these sites deletes exons 2 and 3, which encode the first four transmembrane helices, and introduces a frameshift, likely creating a molecular null allele for *dmsr-1. dmsr-1(qn45)* carries a deletion in exon 4 that affects the last three transmembrane helices and causes a frameshift,^12^ whereas *dmsr-1(sy1522)* contains a CRISPR-Cas9-engineered STOP-IN cassette in the first transmembrane helix, located in exon 2.^20^ Therefore, all three *dmsr-1* alleles are predicted to be strong loss-of-function or null alleles. We backcrossed all three *dmsr-1* alleles into the same N2 background and measured sleep in all three strains simultaneously using the same microfluidic chip. All three alleles caused the same strong reduction in sleep, indicating that recombination of *dmsr-1[FRT exons 2-3 FRT]* results in a strong loss-of-function or null phenotype. The three alleles are not statistically significantly different (*p* > 0.05, Wilcoxon rank-sum test). Sleep reduction in all alleles is due to shorter and less-frequent sleep bouts. N2: *n* = 48, *dmsr-1[FRT]*: *n* = 62, *dmsr-1(sy1522)*: *n* = 64, *dmsr-1(qn45)*: *n* = 63. n.s. = not significant (*p* > 0.05), *p ≤ % 0.05, ****p ≤ % 0.0001, Wilcoxon rank-sum test. (C) Cell-specific knockdown of *dmsr-1* across major neuronal subpopulations. The number of animals used for each genotype is indicated in the graph. The number of animals used is displayed in the figure. Biological replicates: cholinergic neuron knockdown: 2, body wall muscle knockdown: 3, dopaminergic knockdown: 1, mechanosensory neuron knockdown: 2, serotoninergic knockdown: 2, glutamatergic knockdown: 2, GABAergic neuron knockdown: 3. n.s. = not significant (*p* > 0.05), *p ≤ % 0.05, **p ≤ % 0.01, ****p ≤ % 0.0001, Wilcoxon rank-sum test. (D) Sleep architecture of *dmsr-1* knockdown in cholinergic neurons. *dmsr-1[FRT exons 2-3 FRT]*: *n* = 23, *unc-17p::FLP*: *n* = 46, *dmsr-1[FRT exons 2-3 FRT]; unc-17p::FLP*: *n* = 41, 2 biological replicates. The knockdown causes both significantly shorter and less-frequent sleep bouts. ****p ≤ % 0.0001, Wilcoxon rank-sum test. Sleep architecture of *dmsr-1* knockdown in GABAergic neurons. *dmsr-1[FRT exons 2-3 FRT]*: *n* = 54, *unc-47::FLP*: *n* = 49, *dmsr-1[FRT exons 2-3 FRT]; unc-47::FLP*: *n* = 45, 3 biological replicates. The knockdown causes a significant increase in bout length without changing bout frequency. n.s. = not significant (*p* > 0.05), ****p ≤ % 0.0001, Wilcoxon rank-sum test. (F and G) Cholinergic *dmsr-1* is required for sleep downstream of RIS. *unc-17p::FLP*: *n* = 25, *dmsr-1[FRT exons 2-3 FRT]*: *n* = 16, *unc-17p::FLP; dmsr-1[FRT exons 2-3 FRT]*: *n* = 23, with 3 biological replicates. The corresponding bout alignment can be found in Figure S3I. (F) Alignment of RIS calcium activity peaks shows no significant changes in RIS transients in the conditional knockdown of *dmsr-1* in cholinergic neurons compared with its controls. However, sleep was reduced by approximately 40% at the RIS transient peak in the knockdown. *p ≤ % 0.05, ***p ≤ % 0.001. Wilcoxon rank-sum test was performed for the average GCaMP intensity and sleep fraction from −1 to 1 min and from 5 to 10 min. (G) The knockdown of *dmsr-1* in cholinergic neurons does not change the frequency of RIS activation peaks. n.s. = not significant (*p* > 0.05), *p ≤ % 0.05, Wilcoxon rank-sum test. (H and I) Aldicarb treatment causes a small reduction in RIS activity and strongly impairs sleep. Wild-type background + vehicle (H_2_O): *n* = 137, wild-type background + 5 mM aldicarb: *n* = 129, with 2 biological replicates for each condition. (H) Aldicarb causes a small reduction in RIS peak frequency, *p ≤ % 0.05, Wilcoxon rank-sum test. (I) Alignment of RIS calcium activity peaks suggests that aldicarb slightly reduces RIS activity, while it decreases sleep during RIS peak activity by approximately 3-fold. ****p ≤ % 0.0001, Wilcoxon rank-sum test for GCaMP intensity and sleep fraction from −1 to 1 min relative to sleep bout onset. See also Figure S3 and Data S1, S2 and S4.

Our analysis revealed that DMSR-1 has dual roles, inducing sleep and inhibiting RIS. Because DMSR-1 is an inhibitory receptor, we hypothesized that these opposing roles might be carried out in different neuronal cell types. Single-cell sequencing has identified 128 transcriptionally distinct neuron types in *C. elegans*, and DMSR-1 is expressed in at least 82 of them.^28^ To pinpoint the neuronal cell types through which DMSR-1 regulates sleep, we performed conditional knock-downs of *dmsr-1* in specific neuronal subpopulations. We engineered a conditional deletion allele of *dmsr-1*, named *dmsr-1 [FRT exons 2-3 FRT]* (Figures 3A and 3B; Data S1), and combined it with transgenic drivers expressing FLP recombinase in major neuronal subpopulations, classified by their neurotransmitter types (Data S2).^14,29^ We then measured the fraction of time spent sleeping in these conditional mutants and their respective controls. When we knocked down *dmsr-1* in cholinergic neurons, it nearly eliminated sleep, reducing both sleep bout frequency and duration (Figures 3C and 3D). Cholinergic transmission is the primary excitatory signaling type in *C. elegans*,^30–32^ and our prior research indicates that persistent diacylglycerol kinase (DGK)-1 signaling in cholinergic neurons prevents sleep.^33,34^ This supports the idea that reducing cholinergic neuron activity is crucial for sleep induction. Although we attempted to pinpoint specific cholinergic subpopulations responsible for this effect, no single population could be identified to be essential (Figures S3A–S3F). In sharp contrast to the cholinergic knockdown, knocking down *dmsr-1* in GABAergic neurons nearly doubled sleep time by extending sleep bout durations (Figures 3C and 3E). Thus, *dmsr-1* plays opposing roles in sleep regulation, in cholinergic neurons it is required for sleep and in GABAergic neurons it inhibits sleep.

To determine whether the sleep phenotypes observed with cell type-specific *dmsr-1* knockdown extend to sleep-related functions, we examined the effects on *hsp-12*.*6* expression. Consistent with the sleep data, knocking down *dmsr-1* in cholinergic neurons reduced *hsp-12*.*6* expression, while knockdown in GABAergic neurons increased it (Figures S3G and S3H). The beneficial role of *dmsr-1* in cholinergic neurons aligns with the observation that excessive cholinergic signaling at neuromuscular junctions impairs health and lifespan by inhibiting FoxO (DAF-16) signaling,^35^ as well as with the finding that sleep loss reduces FoxO-dependent *hsp-12*.*6* expression and survival.^33^

### Cholinergic signaling sustains wakefulness downstream of RIS activation

**Figure 4:**
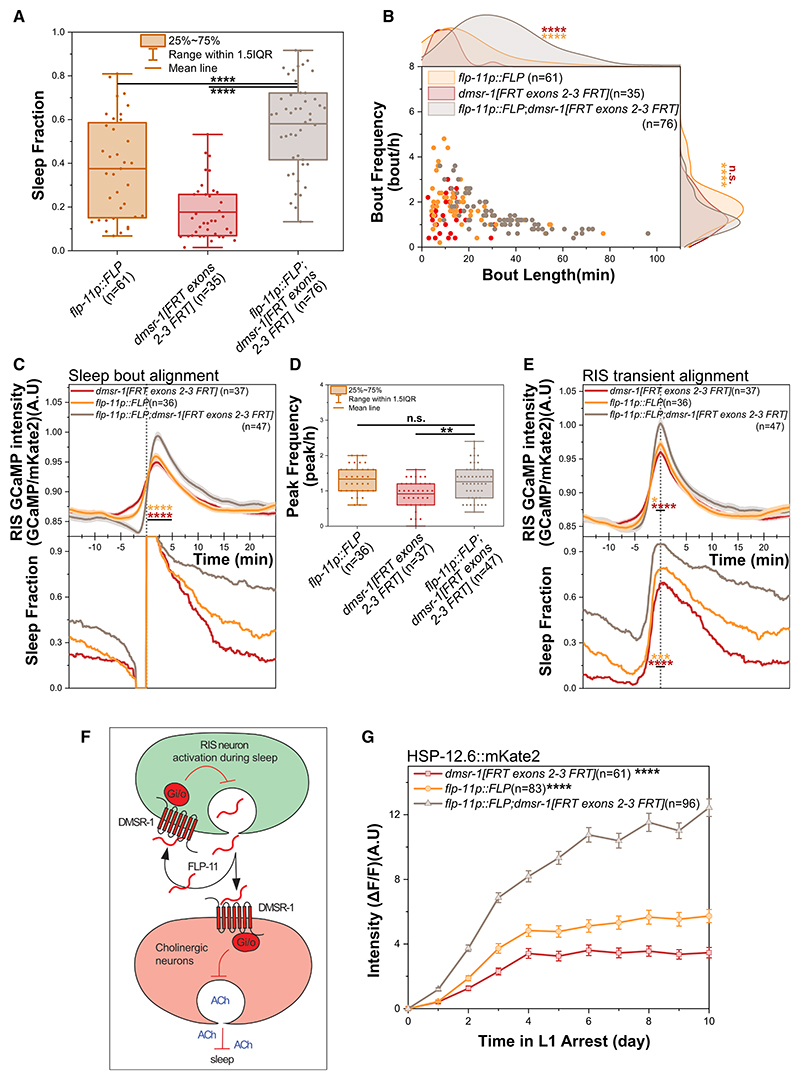
DMSR-1 acts in RIS to inhibit its activation and limit sleep duration (A and B) *dmsr-1* knockdown in RIS, driven by FLP expression from the *flp-11* promoter, increases sleep. *flp-11p::FLP*: *n* = 61, *dmsr-1[FRT exons 2-3 FRT]*: *n* = 35, *flp-11p::FLP; dmsr-1[FRT exons 2-3 FRT]*: *n* = 76, with 3 biological replicates. (A) The fraction of time spent sleeping increased significantly upon knockdown of *dmsr-1* in RIS. ****p ≤ % 0.0001, Wilcoxon rank-sum test. (B) *dmsr-1* knockdown in RIS causes longer sleep bouts without affecting the frequency of the bouts. n.s. = not significant (*p* > 0.05), ****p ≤ % 0.0001, Wilcoxon rank-sum test. (C–E) *dmsr-1* knockdown in RIS increases RIS activity during sleep and causes stronger and longer RIS activation transients, although the frequency of RIS activation peaks remains unchanged. *flp-11p::FLP*: *n* = 37, *dmsr-1[FRT exons 2-3 FRT]*: *n* = 36, *flp-11p::FLP; dmsr-1[FRT exons 2-3 FRT]*: *n* = 47, with 3 biological replicates. *p ≤ % 0.05, **p ≤ % 0.01, ****p ≤ % 0.0001, Wilcoxon rank-sum test. (C) Sleep bout alignment reveals increased RIS activity during sleep. Statistical testing was performed for the 5 min after sleep onset. ****p ≤ % 0.0001, Wilcoxon rank-sum test for GCaMP intensity from 0 to 5 min. (D) RIS activation peak frequency is not significantly changed. n.s. = not significant (*p* > 0.05), **p ≤ % 0.01, Wilcoxon rank-sum test. (E) RIS activity peak alignment shows stronger RIS calcium activity and increased sleep at the peak maximum. *p ≤ % 0.05, ***p ≤ % 0.001, ****p ≤ % 0.0001. Wilcoxon rank-sum test for averaged GCaMP intensity and sleep fraction per worm from −1 to 1 min. (F) Model of how RIS-released FLP-11 signals through DMSR-1. At the onset of the sleep bout, RIS secretes FLP-11, which binds to DMSR-1 in cholinergic neurons. This binding activates G_i/o_, thereby limiting the release of acetylcholine and inducing sleep. Additionally, FLP-11 also binds to DMSR-1 in RIS, reducing RIS activity and the duration of sleep. *(G) dmsr-1* knockdown in RIS increases the expression of *hsp-12*.*6. flp-11p::FLP*: *n* = 83, *dmsr-1[FRT exons 2-3 FRT]*: *n* = 61, *flp-11p::FLP; dmsr-1[FRT exons 2-3 FRT]*: *n* = 96, with 3 biological replicates. ****p ≤ 0.0001, The Wilcoxon rank-sum test was performed by averaging the fluorescence intensity per worm over the last 4 days. See also Figure S4 and Data S2, S4 and S4.

*dmsr-1* knockdown in cholinergic neurons could impair sleep by either inhibiting RIS activation or by acting independently of RIS, i.e., downstream of RIS activity. To test the effect of cholinergic knockdown on RIS activation, we imaged GCaMP in this neuron. Sleep bout and RIS peak alignment analyses did not reveal any differences in RIS activation. The frequency and strength of RIS activation peaks were normal. However, sleep during RIS activation was reduced (Figures 4F, 4G, and S3I). These results support the model that *dmsr-1* expression in cholinergic neurons is required for sleep downstream of RIS activation, consistent with the idea that RIS-released FLP-11 inhibits cholinergic neurons during sleep.

The significant reduction in sleep observed in the cholinergic neuron knockdown of *dmsr-1* suggests that RIS inhibition of cholinergic transmission is essential for sleep. To test this hypothesis, we used aldicarb at a low concentration that pharmacologically slows the breakdown of released acetylcholine without inducing paralysis^36^ and examined its effects on sleep and RIS activity. RIS calcium transient alignment revealed a minimal reduction in transient frequency and strength, while the fraction of worms that slept during RIS activation was strongly reduced in the presence of aldicarb (Figures 3H, 3I, and S3J–S3L). These findings support the idea that reducing cholinergic signaling downstream of RIS is crucial for sleep induction.

### DMSR-1 acts in RIS to inhibit its activation and limit sleep duration

We previously demonstrated that knocking down *dmsr-1* in GABAergic neurons increases sleep. The GABAergic FLP re-combinase driver expresses in seven different cell types, among which only the RMEV/Ds and RIS neurons are known to express *dmsr-1*.^28^ To determine whether *dmsr-1* acts in RME or RIS neurons to inhibit sleep, we generated driver lines that expressed FLP recombinase either in RME neurons or in the RIS neuron. For targeting RME neurons, we used a fragment of the *unc-25* promoter,^37^ and for RIS, we used both the *flp-11*^8,28^ and *srx-9*^28^ promoters. We validated the specificity of these driver lines using a fluorescent protein expression readout.^14^ The RME FLP recombinase driver line caused recombination in RME neurons, although the recombination was not highly penetrant (Figure S4A). The *flp-11p::FLP* driver consistently caused recombination in RIS, as well as in several other cell types. The *srx-9::SL2::FLP* driver caused recombination in RIS in most of the individuals, with no recombination detected outside of RIS (Figure S4B).

The conditional *dmsr-1* knockdown in the RMEs did not affect the time spent sleeping (Figure S4C). However, due to the inefficiency of recombination in the RMEs, we cannot exclude them as a site of *dmsr-1* action. In contrast, both conditional *dmsr-1* knockdowns in RIS resulted in a significant increase in sleep (Figures 4A, 4B, S4D, and S4E). These findings indicate that *dmsr-1* is required in RIS to inhibit sleep.

The inhibition of sleep by *dmsr-1* in RIS suggested that *dmsr-1* might also inhibit calcium activation in this neuron. To test this hypothesis, we imaged RIS calcium activity throughout the sleep-wake cycle when *dmsr-1* was knocked down in RIS. We quantified sleep and RIS calcium activity, aligning the data with both sleep bout onset and the peak of RIS activation. We used both recombinase drivers, with the *flp-11* promoter-driven driver causing a stronger phenotype. Knockdown of *dmsr-1* with either FLP recombinase driver line did not affect the frequency of RIS peaks. However, there was an increase in the size and duration of RIS activation peaks (Figures 4C–4E and S4F–S4H). Thus, *dmsr-1* in RIS limits sleep duration by inhibiting RIS calcium activity. These results suggest a homeostatic autocrine negative feedback model in which sleep induction is coupled with the limitation of sleep duration, effectively preventing excessive sleep (Figure 4F).

To assess the functional consequences of increased RIS activity and sleep following *dmsr-1* knockdown in RIS, we quantified *hsp-12*.*6* expression and L1 arrest survival. Consistent with increased sleep, we observed higher *hsp-12*.*6* expression (Figures 4G and S4I). Although increasing sleep thus has a beneficial role, excessive sleep could hypothetically also increase vulnerability to predation and lead to opportunity costs, making it potentially evolutionarily maladaptive.

## Discussion

Here, we show that, in addition to ALA-released FLP-13,^4,9,12,25,38^ RIS-released FLP-11 activates DMSR-1. Thus, the two key *C. elegans* sleep neurons use different neuropeptides that converge on the promiscuous DMSR-1 receptor. Whether ALA-released FLP-13 also acts on DMSR-1 in cholinergic neurons to promote sleep is an intriguing question for future research. Among the 128 transcriptionally distinct neuronal types, at least 75 express the acetylcholine transporter gene *unc-17*, and the majority of these (at least 68) also express *dmsr-1*.^28^ Among the neuronal cell types that express both *dmsr-1* and *unc-17*, only four are postsynaptic to RIS,^28,39^ suggesting that FLP-11 may act on DMSR-1 via both synaptic and extrasynaptic transmission.^40,41^ This suggests that multiple cholinergic neuron types may need to be inhibited to generate sleep.

ALA also expresses *dmsr-1*,^28^ raising the possibility of an additional autoregulatory feedback mechanism in which ALA releases FLP-13 to also inhibit itself. Speculatively, RIS and ALA could not only both inhibit themselves but also each other.

Why do ALA and RIS use different RFamide neuropeptides to regulate sleep through DMSR-1? The neuropeptides FLP-11 and FLP-13 are both promiscuous themselves. Each activate a set of overlapping receptors, including DMSR-1, DMSR-7, and FRPR-8. However, they also target distinct GPCRs. FLP-13 activates FRPR-4, while FLP-11 activates NPR-22.^13^ Thus, FLP-13 and FLP-11 have some overlapping but also divergent receptor targets. The promiscuity of DMSR-1 might thus allow ALA and RIS to use different neuropeptides with overlapping but distinct targets, enabling them to perform their distinct yet related roles in regulating sleep.^8,11,17–19,23,25,38,42–47^

DMSR-1 is an ortholog of the *Drosophila* myosuppressin receptor.^48^ It belongs to a supergroup of protostome peptide GPCRs related to RFamide and Wamide-activated receptors, which form a protostome sister clade to the deuterostome GPR139 and GPR142 receptors.^49^ Application of a GPR139 agonist increases non-rapid eye movement (NREM) sleep duration in a dose-dependent manner without affecting rapid eye movement (REM) sleep. GPR139 knockout mice spend less time in REM sleep during the dark phase compared with their wild-type littermates, while NREM sleep remains unchanged.^50^ This suggests that GPR139 plays a role in supporting sleep. Given our findings with DMSR-1, it would be valuable to use conditional GPR139 knockdowns in mice to determine whether GPR139 acts in wake-fulness circuits to support sleep and whether it additionally acts in sleep-active neurons to limit sleep. Based on our results in *C. elegans*, it is likely that mammalian sleep is regulated by similar RFamide signaling pathways, which control sleep induction by inhibiting wakefulness neurons and regulate sleep duration through the self-inhibition of sleep-active neurons.

## Resource Availability

### Lead contact

Requests for further information, resources, and reagents should be directed to and will be fulfilled by the lead contact, Henrik Bringmann (henrik.bringmann@tu-dresden.de).

### Materials availability

Key *C. elegans* strains that were used in this project are available at the Caenorhabditis Genetics Center. Other *C. elegans* strains or additional reagents generated for this study are available upon reasonable request from the lead contact.

## Star⋆Methods

Detailed methods are provided in the online version of this paper and include the following:

- Key resources table
- Experimental model and subject details
  - *C. elegans*
  - *Xenopus laevis*
- Method details
  - *C. elegans* methods
  - Aldicarb treatment
  - Heat shock for *flp-11* overexpression
  - *Xenopus laevis* methods
- Quantification and statistical analysis
  - Sleep detection based on quantification of movement quiescence
  - Motion detection using DIC imaging
  - Motion detection using calcium imaging data
  - Scoring of sleep from motion data B Functional Ca^2+^ imaging analysis B RIS intensity extraction
  - Sleep onset alignment
  - RIS peak alignment
  - Quantification of HSP-12.6::mKate2 expression
  - Sample size and number of replicates

## Star⋆Methods

**Table:** Key Resources Table

REAGENT or RESOURCE	SOURCE	IDENTIFIER
Bacterial and virus strains		
*Escherichia coli*	CGC	OP50
Chemicals, peptides, and recombinant proteins		
Aldicarb	Chemodex	A0320
Deposited data		
Raw and analyzed data	Mendeley data	https://doi.org/10.17632/jfpmmdfx6p.1
Experimental models: *C. elegans* strains		
Wild type	CGC	N2
*dmsr-1(qn45) V.*	This study, obtained by backcrossing NQ915,^12^ gift of David Raizen (backcrossed 6 x)	HBR1373
*dmsr-1(qn45) V.*	This study, obtained by backcrossing HBR1373 (backcrossed 6 x)	HBR3762
*dmsr-1(sy1522) V.*	CGC (backcrossed 0 x)	PS8789
*dmsr-1(sy1522) V.*	This study, obtained by backcrossing PS8789 (backcrossed 6 x)	HBR3763
*goeIs304[flp-11p∷SL1-GCaMP3.35-* *SL2∷mKate2-unc-54-3’UTR, unc-119(+)].*	Wu et al.^17^ (backcrossed 2 x)	HBR1361
*dmsr-1(qn45) V; goeIs304[flp-11p∷SL1-* *GCaMP3.35-SL2∷mKate2-unc-54-3’UTR, unc-119(+)].*	This study (backcrossed 0 x)	HBR2727
*dmsr-7(syb2457) V.*	This study (backcrossed 0 x)	PHX2457
*frpr-8(db1233) X.*	de Bono lab (provided by Isabel Beets) (backcrossed 0 x)	AX7163
*npr-22(ok1598) X; npr-4(tm1782) X; frpr-3(ok3302) V.*	Turek et al.^8^ (backcrossed 0 x)	HBR1300
*flp-11(tm2706) X.*	Turek et al.^8^ (backcrossed 9 x)	HBR507
*dmsr-1(qn45) V; flp-11(tm2706) X.*	This study (backcrossed 0 x)	HBR2826
*goe!s240[hsp16.2p:^lp-11∷SL2∷* *mKate2∷unc-54-3UTR, unc-119(+)].*	Turek et al.^8^ (backcrossed 2 x)	HBR1021
*dmsr-1(qn45) V; goeIs240[hsp 16.2p∷flp-11∷* *SL2∷mKate2∷unc-54-3UTR, unc-119(+)].*	This study (backcrossed 0 x)	HBR1492
*syb2346syb2493[flp-11p∷ReaChR∷Linker∷ mKate2∷flp-11 3’UTR]) III:7007600; goeIs304 [flp-11p∷SL1-GCaMP3.35∷SL2∷mKate2∷ unc-54-3’UTR, unc-119(+)].*	Busack and Bringmann^18^ (backcrossed 0 x)	HBR2523
*syb2346syb2493[flp-11p∷ReaChR∷Linker∷* *mKate2∷flp-11 3’UTR]) III:7007600;* *goeIs304[flp-11p∷SL1-GCaMP3.35-* *SL2∷mKate2∷unc-54∷3’UTR,* *unc-119(+)]; dmsr-1(qn45)V.*	This study (backcrossed 0 x)	HBR2764
*hsp-12.6(syb1364[hsp-12.6∷mKate2]) IV.*	Koutsoumparis et al.^33^ (backcrossed 0 x)	PHX1364
*hsp-12.6(syb1364[hsp-12.6∷mKate2]) IV; dmsr-1(qn45) V.*	This study (backcrossed 0 x)	HBR2824
*flp-11(syb5866) X.*	This study (backcrossed 0 x)	PHX5866
*flp-25(yum424) III.*	This study (backcrossed 1 x), the alle was a gift from Changchun Chen, Pu et al.^24^	HBR3269
*flp-13(tm2427) IV.*	National Bioresource Project (backcrossed 0 x)	FX02427
*dmsr-1(syb6331syb6333[FRT∷dmsr-1 exons 2-3∷FRT]) V.*	This study (backcrossed 0 x)	PHX6333
*dmsr-1(syb6331syb6333[FRTI) V.*	This study, via germline recombination of PHX6333 (backcrossed 0 x)	HBR3477
*dmsr-1(syb6331syb6333[FRTI) V.*	This study, obtained backcrossing HBR3477 (backcrossed 5 x)	HBR3764
*bqSi294[pBN154(unc-119(+) hsp16.41p>mCh∷ his-58>gfp∷his-58)] II; bqSi1123[pBN515(unc-119(+) unc-17p∷FLP∷SL2∷mNG)] IV.*	Gift from Peter Askjaer, Fragoso et al.^29^ (backcrossed 0 x)	BN1125
*bqSi1123[pBN515(unc-119(+)* *unc-17p∷FLP∷SL2∷mNG)] IV; dmsr-1* *(syb6331syb6333[FRT∷dmsr-1 exons 2-3∷FRT]) V.*	This study (backcrossed 0 x)	HBR3078
*bqSi294[hsp16.41p∷FRT∷mCherry∷his-58∷FRT∷* *GFP∷his-58 + unc-119(+)] II; bqSi495 [myo-3p∷ FLP∷D5 + unc-119(+)] IV.*	CGC (backcrossed 2 x)	BN503
*bqSi495[myo-3p∷FLP∷D5 + unc-119(+)] IV;* *dmsr-1(syb6331syb6333[FRT∷dmsr-1 exons 2-3∷FRT])V.*	This study (backcrossed 0 x)	HBR2991
*bqSi294[hsp16.41p∷FRT∷mCherry∷his-58∷FRT∷GFP∷his-58 + unc-119(+)] II; bqSi614 [dat-1p∷FLP D5∷SL2∷mNG + unc-119(+)] IV.*	CGC (backcrossed 2 x)	BN617
*bqSi614[dat-1p∷FLP D5∷SL2∷mNG + unc-119(+)] IV; dmsr-1(syb6331syb6333[FRT∷ dmsr-1 exons 2-3∷FRT]) V.*	This study (backcrossed 0 x)	HBR3080
*bqSi294[hsp16.41p∷FRT∷mCherry∷his-58∷* *FRT∷GFP∷his-58 + unc-119(+)] II; bqSi487[mec-7p∷FLP D5 + unc-119(+)] IV.*	CGC (backcrossed 2 x)	BN498
*bqSi487[mec-7p∷FLP D5 + unc-119(+)] IV;* *dmsr-1(syb6331syb6333[FRT∷dmsr-1 exons 2-3∷FRT]) V.*	This study (backcrossed 0 x)	HBR3029
*bqSi294[hsp16.41p∷FRT∷mCherry∷his-58∷FRT∷GFP∷his-58 + unc-119(+)] II; bqSi488[tph-1p∷FLP D5 + unc-119(+)] IV.*	CGC (backcrossed 2 x)	BN499
*bqSi488[tph-1p∷FLP D5 + unc-119(+)] IV;* *dmsr-1(syb6331syb6333[FRT∷dmsr-1 exons 2-3∷FRT]) V.*	This study (backcrossed 0 x)	HBR2999
*bqSi294[pBN154(unc-119(+) hsp16.41p∷* *FRT∷mCh∷his-58∷FRT∷gfp∷his-58)] II;* *bqSi991[pBN471(unc-119(+) eat-4p∷* *FLP∷SL2∷mNG)] IV.*	Gift from Peter Askjaer and Emanuel Busch (backcrossed 0 x)	BN993
*bqSi991[pBN471(unc-119(+) eat-4p∷FLP∷* *SL2∷mNG)] IV; dmsr-1(syb6331syb6333[FRT∷ dmsr-1 exons 2-3∷FRT]) V.*	This study (backcrossed 0 x)	HBR3081
*bqSi294[unc-119(+) hsp-16.41p∷FRT∷mCh∷ his-58∷FRT∷gfp∷his-58] II; bqSi542[unc-47p∷ FLPD5 + unc-119(+)].*	CGC (backcrossed 2 x)	BN544
*bqSi542[unc-47p∷FLP D5 + unc-119(+)] IV;* *dmsr-1(syb6331syb6333[FRT∷dmsr-1 exons 2-3∷FRT]) V.*	This study (backcrossed 0 x)	HBR2992
*nmr-1(syb8025[nmr-1∷SL2∷FLP D5]) II.*	This study (backcrossed 0 x)	PHX8025
*nmr-1(syb8025[nmr-1∷SL2∷FLP D5]) II;* *dmsr-1(syb6331syb6333[FRT∷dmsr-1 exons 2-3∷FRT]) V.*	This study (backcrossed 0 x)	HBR3318
*bqSi294[hsp16.41p∷FRT∷mCherry∷his-* *58∷FRT∷GFP∷his-58 + unc-119(+)] II.*	This study (backcrossed 0 x) from BN499	HBR2998
*unc-4(syb9007[unc-4∷SL2∷FLP D5]) II.*	This study (backcrossed 0 x)	PHX9007
*unc-4(syb9007[unc-4∷SL2∷FLP D5])II, bqSi294 [hsp16.41p∷FRT∷mCherry∷his-58∷FRT∷GFP∷his-58 + unc-119(+)] II.*	This study (backcrossed 0 x)	HBR3518
*unc-4(syb9007[unc-4∷SL2∷FLP D5]) II; dmsr-1(syb6331syb6333[FRT∷dmsr-1 exons 2-3∷FRT]) V.*	This study (backcrossed 0 x)	HBR3519
*bqSi542[unc-47p∷FLP D5 + unc-119(+)], hsp-12.6(syb1364[hsp-12.6∷mKate2]) IV; dmsr-1(syb6331syb6333[FRT∷dmsr-1 exons 2-3∷FRTI) V.*	This study (backcrossed 0 x)	HBR3083
*hsp-12.6(syb1364[hsp-12.6∷mKate2]) IV;* *dmsr-1(syb6331syb6333[FRT∷dmsr-1 exons 2-3∷FRTI) V.*	This study (backcrossed 0 x)	HBR3084
*bqSi542[unc-47p∷FLP D5 + unc-119(+)], hsp-12.6(syb1364[hsp-12.6∷mKate2]) IV.*	This study (backcrossed 0 x)	HBR3085
*hsp-12.6(syb1364[hsp-12.6∷mKate2]) IV, bqSi1123[pBN515(unc-119(+) unc-17p∷FLP∷SL2∷mNG)] IV;* *dmsr-1(syb6331syb6333[FRT∷ dmsr-1 exons 2-3∷FRT]) V.*	This study (backcrossed 0 x)	HBR3169
*hsp-12.6(syb1364[hsp-12.6∷mKate2]) IV, bqSi1123[pBN515(unc-119(+) unc-17p∷FLP∷SL2∷mNG)] IV.*	This study (backcrossed 0 x)	HBR3170
*dmsr-1(syb6331syb6333[FRT∷dmsr-1* *exons 2-3∷FRT&iexcl;) V; goeIs304[flp-11p∷* *SL1-GCaMP3.35-SL2∷mKate2∷unc-* *54∷3’UTR, unc-119(+)].*	This study (backcrossed 0 x)	HBR3388
*bqSi1123[pBN515(unc-119(+)* *unc-17p∷FLP∷SL2∷mNG)] IV;* *dmsr-1(syb6331syb6333[FRT∷dmsr-1* *exons 2-3∷FRT]) V; goeIs304[flp-11p∷* *SL1-GCaMP3.35-SL2∷mKate2∷* *unc-54∷3’UTR, unc-119(+)].*	This study (backcrossed 0 x)	HBR3387
*bqSi1123[pBN515(unc-119(+)* *unc-17p∷FLP∷SL2∷mNG)] IV;* *goeIs304[flp-11p∷SL1-GCaMP3.35-* *SL2∷mKate2∷unc-54∷3’UTR, unc-119(+)].*	This study (backcrossed 0 x)	HBR3389
*syb2346syb6265[flp-11p∷FLP* *D5∷flp-11 3’UTR] III:7007600.*	This study (backcrossed 0 x)	PHX6265
*syb2346syb6265[flp-11p∷FLP* *D5∷flp-11 3’UTR] III:7007600;* *(syb6331syb6333[FRT∷dmsr-1* *exons 2-3∷FRTI) V.*	This study (backcrossed 0 x)	HBR3431
*syb2346syb6265[flp-11p∷FLP* *D5∷flp-11 3’UTR] III:7007600;* *dmsr-1(syb6331syb6333[FRT∷dmsr-1* *exons 2-3∷FRT]) V; goeIs304[flp-11p∷* *SL1-GCaMP3.35-SL2∷mKate2∷* *unc-54∷3’UTR, unc-119(+)].*	This study (backcrossed 0 x)	HBR3452
*syb2346syb6265[flp-11 p∷FLP* *D5∷flp-11 3’UTR] 111:7007600;* *goeIs304[flp-11p∷SL1-GCaMP3.35-* *SL2∷mKate2∷unc-54∷3’UTR, unc-119(+)].*	This study (backcrossed 0 x)	HBR3453
*syb2346syb6265[flp-11 p∷FLP D5∷flp-11 3’UTR] III:7007600; hsp-12.6 (syb1364[hsp-12.6∷mKate2]) IV;* *dmsr-1(syb6331syb6333[FRT∷dmsr-1 exons 2-3∷FRT]) V.*	This study (backcrossed 0 x)	HBR3474
*syb2346syb6265[flp-11 p∷FLP* *D5∷flp-11 3’UTR] III:7007600;* *hsp-12.6(syb1364[hsp-12.6∷mKate2]) IV.*	This study (backcrossed 0 x)	HBR3475
*syb812 7[dpy-10site∷unc-25 fragment with tataa sites∷ dpy-10site∷SL1-aaaa∷FLP D5∷let-858 3’utr] IV:5015000.*	This study (backcrossed 0 x)	PHX8127
*bqSi294[hsp16.41p∷FRT∷* *mCherry∷his-58∷FRT∷GFP∷* *his-58 + unc-119(+)] II; syb8127[dpy-10site∷unc-25 fragment with tataa sites∷dpy-10site∷SL1-aaaa∷FLP D5∷let-858 3’utr] IV:5015000.*	This study (backcrossed 0 x)	HBR3353
*srx-9(syb7929[srx-9∷SL2∷FLP D5]) V.*	This study (backcrossed 0 x)	PHX7929
*bqSi294[hsp16.41p∷FRT∷mCherry∷* *his-58∷FRT∷GFP∷his-58 + unc-119(+)] II;* *srx-9(syb7929[srx-9∷SL2∷FLP D5]) V.*	This study (backcrossed 0 x)	HBR3236
*dmsr-1(syb6331syb6333[FRT∷dmsr-1 exons 2-3∷FRT]) V, srx-9(syb7929[srx-9∷ SL2∷FLP D5]) V.*	This study (backcrossed 0 x)	HBR3430
*dmsr-1(syb6331syb6333[FRT∷dmsr-1 exons 2-3∷FRT]), srx-9(syb7929[srx-9∷ SL2∷FLPD5]) V; goeIs304[flp-11p∷SL1-GCaMP3.35-SL2∷mKate2∷unc-54∷3’UTR, unc-119(+)].*	This study (backcrossed 0 x)	HBR3450
*srx-9(syb7929[srx-9∷SL2∷FLP D5]) V;* *goeIs304[flp-11p∷SL1-GCaMP3.35-SL2∷* *mKate2∷unc-54∷3’UTR, unc-119(+)].*	This study (backcrossed 0 x)	HBR3451
*hsp-12.6(syb1364[hsp-12.6∷mKate2]) IV; dmsr-1(syb6331syb6333[FRT∷dmsr-1 exons 2-3∷FRT]), srx-9(syb7929[srx-9∷SL2∷FLP D5]) V.*	This study (backcrossed 0 x)	HBR3472
*hsp-12.6(syb1364[hsp-12.6∷mKate2]) IV;* *srx-9(syb7929[srx-9∷SL2∷FLP D5]) V.*	This study (backcrossed 0 x)	HBR3473
	*When the information about backcrosses was not available, we indicated as 0 x	
Oligonucleotides for genotyping		
Target allele	Forward	Reverse
*dmsr-1(qn45)*	TCGTGATCGTCACCCTAGC; CAACATCGACCACCGAATCAAACG	GCAATTGTCGGAAAATGTAGAG
*dmsr-1(sy122)*	GTAAGTTTTACTTTTGTGTCTC	CTTAGTCACCTCTGCTCTG
*flp-11(tm2706)*	TCTTCCAAATCGAACCAAGG; ATGATGAATTCGCCTCAGGA	TAGCCGCTCGTCTCACTTTT
*syb2346syb2493[flp-11 p∷ReaChR∷* *linker∷mKate2∷flp-11 3’UTR]*	AGACCACCTACCGTTCCAAG; ATGGCGATGTCATTTTCATGTT	ATCCCAGTTGTTTGACGGTT
*dmsr-1[FRT exons 2-3 FRT]*	GTAAGTTTTACTTTTGTGTCTC; AAGTTCCTATTCTCTAGAAAGTATAG	TTAACAAAAATCTTGTTTTTTTTTTAC; AAGGTTTATATAAATCCAAAGC
*bqSi1123[pBN515(unc-119(+) unc-17p∷* *FLP∷SL2∷mNG)], bqSi495 [myo-3p∷* *FLP∷D5 + unc-119(+)], bqSi614 [dat-1p∷ FLP D5∷SL2∷mNG + unc-119(+)], bqSi487 [mec-7p∷FLP D5 + unc-119(+)], bqSi488 [tph-1p∷FLP D5 + unc-119(+)],bqSi991 [pBN471(unc-119(+) eat-4p∷FLP∷SL2∷mNG)], bqSi542[unc-47p∷FLP D5 + unc-119(+)]*	TGAAATCATCCCTTGTTGGGAG; GACCTTCCAATCCGCCATATC	TGATTCCCTCTCAAAGCTGC
*nmr-1(syb8025[nmr-1 ∷SL2∷FLP D5])*	TGCTCTCCACTTCCTTGCTTT; TGACCTCCTTCCTCTCCATGA	ACCGTTCCCATTTACTTGCTCA
*unc-4(syb9007[unc-4∷SL2∷FLP D5])*	GCATTGACTACGGTTGCTGA; TGACCTCCTTCCTCTCCATGA	CACGCATTGTGATGCCTACG
*syb2346syb6265[flp-11p∷FLP D5∷flp-11 3’ UTR]*	ATGGCGATGTCATTTTCATGTT; AGTCCTTCAAGCTCGTCCAA	ATCCCAGTTGTTTGACGGTT
*syb8127[dpy-10site∷unc-25 fragment with tataa sites∷dpy-10site∷SL1-aaaa∷FLP D5∷let-858 3’utr]*	GGTGTCCGGCTTTAAATCCAAT; TGACCTCCTTCCTCTCCATGA	ACGATTATTAACAAAATGTCGCCT
*srx-9(syb7929[srx-9∷SL2∷FLPD5])*	CATCAAGTGGGGGAAATAAG; CCACTCGTCTACCTCGACGA	ACACCCGCACATTCATGTAC
Software and algorithms		
NIS advanced Research 5.42.06	Nikon Instrument	https://www.microscope.healthcare.nikon.com/products/software/nis-elements/nis-elements-advanced-research
JupyterLab 4.0.8	Project Jupyter	https://jupyterlab.readthedocs.io/en/latest/
Affinity designer 1.10.5.1342	Serif	https://affinity.serif.com/en-us/designer/?srsltid=AfmBOooNOBJ6nichjJpCg6suLhjut46zTcnhAYqMw_gFavdD-hLHGAg
Algorithms for data analysis	GitHub	https://doi.org/10.5281/zenodo.15025114

## Experimental Model and Subject Details

### C. elegans

We grew *C. elegans* hermaphrodites at 20°C on Nematode Grown Medium (NGM) seeded with the *Escherichia coli* strain OP50.^51^

### Xenopus laevis

Defolliculated *Xenopus laevis* oocytes were purchased from EcoCyte Bioscience and kept in ND96 solution (96 mM NaCl, 1 mM MgCl_2_, 5 mM HEPES, 1.8 mM CaCl_2_, 2 mM KCl) at 4°C until RNA injection.

## Method Details

### *C. elegans* methods

#### Crossing C. elegans strains

To generate males, we heat-shocked young adult worms for 6 hours at 32°C inside an incubator and collected the resulting males in the F1 generation. For strains with phenotypically selectable traits, we generated males by crossing young adult worms with N2 males. We selected homozygous worms by genotyping single worms from the F2 or successive generations using duplex PCR genotyping.^52^ A complete list of strains and the primers used are listed in the key resources table.

### Creation of transgenic worms using the CRISPR/Cas9 system

We designed the transgenes and modifications of the respective endogenous genetic loci *in silico* and had the gene edits performed via CRISPR/Cas9 by an external company (SunyBiotech). We then verified the inserted transgenes and edited endogenous loci through Sanger sequencing. The sequences of the edits can be found in Data S3.

PHX2457 ⟶ *dmsr-7(syb2457) V*.

To generate the allele *syb2457*, the entire coding region of *dmsr-7* was deleted.

PHX5866 ⟶ *flp-11(syb5866) X*.

For *flp-11*, we used a previously characterized deletion allele, *flp-11(tm2706)*, which removes all four peptides encoded by this gene.^8^ We also generated a new allele, *flp-11(syb5866)*, which deletes the entire coding region. To generate the allele *syb5866*, the entire coding region of *flp-11* was deleted.

PHX6333 ⟶ *dmsr-1(syb6331syb6333[FRT::dmsr-1 exons 2-3::FRT]) V*.

The *dmsr-1* conditional allele (*dmsr-1(syb6331syb633)*) was created through the consecutive insertion of two FRT sites^14^ (GAAGTTCCTATTCTCTAGAAAGTATAGGAACTTC) flanking the second and third exons. The sequence within the two *FRT* sites is predicted to encode the first four transmembrane alpha helices. FLP recombination excises this sequence and introduces a frame-shift, resulting in a likely molecular null or strong loss-of-function allele.

**PHX8025 ⟶ *nmr-1(syb8025[nmr-1::SL2::FLP D5]) II*.**

**PHX9007 ⟶ *unc-4(syb9007[unc-4::SL2::FLP D5]) II*.**

**PHX7929 ⟶ *srx-9(syb7929[srx-9::SL2::FLP D5]) V*.**

A codon-optimized sequence of the FLP D5 recombinase was used.^14,53^ This optimized DNA sequence was kindly provided by P. Askjaer. For the alleles *syb8025, syb9007*, and *syb7929*, the optimized coding sequence of the FLP recombinase was inserted after the stop codon of the respective gene, separated by the SL2 splicing site of the *gpd-2*/*gpd-3* operon.^54^

**PHX6265 ⟶ *syb2346syb6265[flp-11p::FLP D5::flp-11 3*’*UTR] III:7007600*.**

*flp-11* is the most strongly expressed gene in RIS but also expresses in other neurons.^8,28^ In contrast, *srx-9* has much lower expression levels but appears to be specific to RIS.^28^ The *flp-11p::FLP D5* driver was generated by integrating the gene encoding FLP recombinase into a single-copy knock-**i**n **l**oci for **d**efined **g**ene **e**xpression (SKI LODGE)^55^ cassette for *flp-11p* expression (*syb2346[flp-11p::dpy-10 site::flp-11 3’UTR] III:7007600*).^18^

**PHX8127 ⟶ *syb8127[dpy-10site::unc-25 fragment with tataa sites::dpy-10site::SL1-aaaa::FLP D5::let-858 3’***UTR***] IV:5015000*.**

To generate the RME::FLP driver, a promoter fragment of *unc-25*, reported to express specifically in the RME neurons,^37^ was used. This fragment, referred to as *prom3del1*, lacks the *unc-30* binding site. TATAA sites were added before this fragment and into the gap where the *unc-30* binding site was, along with an SL1 splicing site. To facilitate future exchanges of the promoter, the promoter was flanked with two *dpy-10* sites. The transgene was inserted at *IV:5015000*, a genetic locus previously shown to allow for transgene expression.^55^

### Characterization of the FLP driver lines

To assess the expression and recombination efficacy of our FLP recombinase driver lines, we used a heat shock-inducible fluorescent reporter as previously described.^14^ Following heat shock, we imaged the worms as detailed below. The worms were immobilized on a microscope slide using a thin layer of 5% agarose (Fisher-Scientific BP164-500) dissolved in M9 buffer. To create the agarose pad, we applied 100 μl of liquid agarose (95°C) and pressed it with a second microscope slide for 10-30 seconds. For immobilization, we pipetted 25 mM Levamisole onto the agarose pad in volumes as specified below, placed the worms into the drop, and covered the pad with a coverslip.

### srx-9::SL2::FLP *characterization*

To investigate the expression of *srx-9::SL2::FLP*, we employed three protocols:

1. Worms were heat shocked on the plate at 32°C for 2 hours, then transferred to 20°C. Eight L1-stage worms were imaged 2 hours post-heat shock.
2. Worms were heat shocked on the plate at 32°C for 2 hours, then transferred to 20°C. Eight L4-stage worms were imaged 3 hours post-heat shock.
3. Worms were heat shocked on the plate at 32°C for 4 hours, then transferred to 20°C. Nine L1-stage worms were imaged 3 hours post-heat shock.

We immobilized the worms using 10 μM Levamisole. In total, 21 out of 25 worms displayed the green signal, as shown in Figure S4. Spinning disc imaging was performed on the TiE setup using a 488 nm laser (0.21 mW/mm^2^, 500 ms exposure) with a 535/30 nm single-band pass filter (Chroma) and a 565 nm laser (0.27 mW/mm^2^, 100 ms exposure) with a 641/75 nm single-band pass filter (Sem-rock). Imaging was conducted through a 100x oil objective, capturing z-stacks with 0.5 μm z-planes over a total distance of 20 μm. In addition to fluorescent images, a widefield z-stack was acquired with a 500 ms exposure. Finally, we computed a maximum intensity projection using NIS software (Nikon).

### nmr-1::SL2::FLP *characterization*

To assess the expression of FLP recombinase in command interneurons, we heat shocked worms at 32°C for 2 h. Afterward, we picked L4 larvae, placed them on imaging slides, and immobilized them using 10 μL of Levamisole. Imaging was performed on the Ti2 microscope with a 550 nm LED (6.05 mW/mm^2^, 60 ms exposure) and a 460 nm LED (8.3 mW/mm^2^, 30 ms exposure), using a triple-band pass filter (LF405/488/594-3X-B-ZHE, Semrock). We also acquired brightfield images using diascopic light with a 7.1 ms exposure. A 100x oil immersion objective was used for imaging, and we captured z-stacks with planes 0.3 μm apart. Maximum intensity projections of the stacks were generated using Fiji (ImageJ).

### unc-4::SL2::FLP *characterization*

To characterize the expression of FLP recombinase in A-type motor neurons, we first grew gravid adult worms. We then released the eggs and cultured them following the protocol outlined in the “survival assay in liquid culture“ section. After 48 hours, we subjected the worms to heat shock at 32°C for 2 hours. L1 arrest larvae were imaged 2 hours post-heat shock. For immobilization, we pipetted 0.3 μL of the liquid culture into a 5 μL drop of Levamisole. Imaging was performed using the Ti2 spinning disk setup with a 100x oil immersion objective. We utilized a 488 nm laser (0.16 mW/mm^2^) with a 525/50 nm single-band pass filter (Semrock), and a 561 nm laser (0.23 mW/mm^2^) with a 641/75 nm single-band pass filter (Semrock), setting the exposure time to 100 ms. Z-stacks were captured with planes 0.2 μm apart, and maximum intensity projections were generated using Fiji (ImageJ).

### RME::FLP characterization

To characterize the expression of FLP recombinase in RME neurons, we heat shocked L4 larvae and young adults on NGM plates at 32°C for 2 hours. Afterward, we placed the worms on imaging slides and immobilized them with 10 μL of Levamisole. Imaging was performed using widefield fluorescence at the Ti2 setup with a 100x oil immersion objective. We used a 460 nm LED (8.3 mW/mm^2^, exposure time 30 ms), a 550 nm LED (6.05 mW/mm^2^, exposure time 60 ms), and a triple-band pass filter (LF405/488/594-3X-B-ZHE, Semrock). Brightfield imaging was also conducted using diascopic light with an exposure time of 7.1 ms. Z-stacks were acquired at 0.3 μm intervals, and maximum intensity projections were generated using Fiji (ImageJ).

### Imaging using microfluidic devices

We conducted long-term imaging by culturing worms in microfluidic devices, as previously described.^21,22^ We created arrays of micrometric square wells (110 x 110 x 10 μm) in agarose using a polydimethylsiloxane (PDMS) mold, which was placed into liquid (95°C) 5% agarose (Fisher Scientific BP164-500) dissolved in M9 buffer. To image worms at the L1 arrest stage, we filled the wells with one pretzel-stage egg each, taking care to avoid transferring any OP50 from the original plate. Eggs from different strains were placed into adjacent wells on the same agarose chip, arranged in recognizable patterns for strain identification under the microscope. We then sealed the wells with a coverslip and glued it into the cut-out hole of a 3.5 cm petri dish, which had its bottom removed to fit the coverslip. The remaining bottom of the dish was filled with agarose, and the gap around the cut-out hole was filled with 5% hot liquid agarose. We sealed the dish with Parafilm M (Sigma-Aldrich) and incubated it upside down at 20°C for 48 hours before imaging. Multiple microchamber arrays were generated and imaged for each experiment on different days, containing individual worms from various NGM plates, corresponding to both biological and technical replicates. Each figure states the number of biological replicates and the number of worms (n). For imaging, the dish was placed into a custom-made sample holder on an inverted microscope and covered with a heating lid set to 25.5°C.

### Microscope setups

We utilized two microscope setups for long-term behavioral imaging, functional Ca^2^+ imaging alone, Ca^2^+ imaging combined with optogenetic stimulation, and fluorescent imaging. 1) The first setup was a Nikon TiE inverted microscope equipped with an automated XY stage (Nikon), a 100W halogen lamp (Osram), and a digital DS-Qi2 camera (SLR, FX-format CMOS sensor, Nikon). 2) The second setup was a Nikon Ti2 inverted microscope featuring an automated XY stage, a diascopic LED, and the fluorescent microscopy illumination LED system CoolLED pE-300^ultra^ (CoolLED), along with a digital Andor camera (back-illuminated sCMOS sensor, 2048 x 2048 pixels, Andor Technology Ltd., Belfast).

We used two additional setups for spinning disk imaging. 1) The first setup was a Nikon TiE inverted microscope equipped with an automated XY stage (Nikon), an EMCCD camera (1024 x 1024 pixels, iXon Ultra 888, Andor), an Andor Revolution disc system (Andor Technology Ltd.), and a Yokogawa CSU-X1 spinning disc head. 2) The second setup consisted of a spinning disk system mounted on a Nikon Ti2 inverted microscope, equipped with an automated XY stage, a digital CMOS camera (2304 x 2304 pixels, ORCA-Fusion BT C15440, HAMAMATSU), and a CSU-W1 Confocal Scanner Unit (YOKOGAWA).

We controlled the imaging experiments on both setups using NIS Element Advanced Research (Nikon) software.

### Differential contrast (DIC) imaging

We conducted long-term DIC imaging using both the TiE and Ti2 setups. In each setup, we used infrared or red filters (Semrock Brightline HC 785/62 and CHROMA 670/50 ET Bandpass, respectively). A 10 x objective lens was employed in both setups to maximize the number of worms per field of view. We imaged the worms after a starvation period of 48 hours, capturing frames at a rate of 0.2 frames per second (FPS) with 2 x 2 binning. In the TiE setup, we maintained constant illumination from the halogen lamp, adjusting the exposure time to between 20 and 40 ms. In the Ti2 setup, the diascopic LED was automatically activated only during exposure, which was set between 4 and 10 ms.

### Functional Ca^2^+ imaging

We conducted all long-term functional Ca^2^+ imaging experiments using the Ti2 setup. We independently controlled the LED channels (460 nm at 0.10 - 0.14 mW/mm^2^ and 550 nm at 0.08 - 0.11 mW/mm^2^) in combination with a triple-band pass filter set (LF405/488/594-3X-B-ZHE, Semrock) for high-speed imaging of fluorescent proteins. To achieve the fastest acquisition speed and minimize exposure of the samples to light that could interfere with sleep behavior, we coordinated the camera’s exposure time and LED exposure via TTL triggering. The exposure time was set between 40 and 60 ms, and images were binned 2 x 2 with a conversion gain of 4. We employed a 20 x objective lens with an additional 1.5 x built-in lens and captured images at a frame rate of 0.17 FPS. In each experiment, we scanned multiple individual fields of view in parallel using an automated stage. We imaged the worms after a 48-hour starvation period for a duration of 5 hours.

### HSP-12.6::mKate2 fluorescence imaging

We measured the expression of the *hsp-12*.*6* gene as described previously. We acquired images of the samples every 24 hours using the Ti2 microscope setup. We utilized 550 nm LED light (6.37–9.23 mW/mm^2^) to excite the fluorescent protein, with an exposure time set at either 60 or 100 ms and a conversion gain of 4. To maximize the number of measurable pixels, we did not use any binning. The samples were imaged using a 20x objective lens and a built-in 1.5x lens. The number of biological replicates for each experiment is specified in the figure legend. When not being imaged, the samples were incubated at 20°C.

### Optogenetic activation

For optogenetic activation experiments, we cultured worms inside agarose microchambers as previously described and imaged them after 48 hours of starvation. Initially, we imaged GCaMP and mKate2 for 6 minutes to record baseline conditions in the absence of retinal, during which ReaChR remained inactive. We then pipetted 10 μL of 10 mM all-trans-retinal (ATR, Sigma Aldrich) dissolved in ethanol onto the agarose containing the microchambers, incubated the samples for an additional 2 hours, and imaged GCaMP and mKate2 again for 6 minutes, while stimulating ReaChR. Imaging was conducted on the Ti2 microscope using a 20x objective and a triple-band pass filter (LF405/488/594-3X-B-ZHE, Semrock). The camera settings included 2x2 binning, a gain of 4, and a frame rate of 0.07 FPS. We used a 460 nm LED (0.14 mW/mm^2^) for GCaMP and a 550 nm LED (0.04 mW/mm^2^) for mKate2, both with an exposure time of 50 ms. To optogenetically stimulate RIS, we exposed the worms to 550 nm LED light (0.03 mW/mm^2^) for 800 ms following each GCaMP frame acquisition. We coordinated the camera’s exposure time and LED exposure using TTL triggering.

### Survival assay in liquid culture

We synchronized the worms by transferring 8-10 L4 larvae onto a seeded NGM plate, which we incubated at 20°C for 4 days. Afterward, we washed the gravid adult worms off the plates with 1 mL of M9 buffer and transferred them into a 2 mL plastic tube. The worms were allowed to settle at the bottom of the tube by gravity for 30 seconds to 2 minutes, after which we removed the supernatant. To release the eggs, we added 500 μL of sodium hypochlorite solution for 30 seconds. We then collected the eggs by centrifuging the sample for 10 seconds using the “short” button on a tabletop centrifuge and discarded the supernatant. After washing the eggs with 1 mL of M9, we recollected them by centrifugation (15 s) and removed the supernatant. We repeated the hypochlorite treatment as before. To stop the bleaching reaction, we washed the eggs three times with 1 mL of M9, recollecting them via centrifugation (30 s) after each wash. Finally, we resuspended the eggs in 1 mL of M9 and transferred the suspension to a fresh 2 mL tube.

We kept the isolated eggs in a 2 mL plastic tube with a total volume of 1 mL of M9, in an incubator at 20°C, on a rotator (VWR) set at 20 rpm for the duration of the experiment. We scored the number of worms that were either dead or alive once per day. For counting, we pipetted an aliquot of the worm suspension (10 μL) onto a seeded NGM plate. To facilitate counting, we allowed the living worms 10-45 minutes at room temperature to recover and spread on the bacterial lawn. We then manually counted the dead and alive worms using a clicker. After the first count on day one, we calculated the population density of worms in the tube and adjusted the number of worms in each vial to 10 worms/μL by adding or removing M9. We kept the plates with the recovered worms in an incubator at 20°C.

Three to four days after recovery, we scored worms that had developed to at least the L4 stage as recovered. The experimenter remained blind to the genotype information during the lifespan experiments. We plotted the curves, smoothing the data points using the adjacent-averaging method in OriginLab software. This method calculates the smoothed value by averaging the data within a moving window of 5 data points. For statistical analysis, we used Fisher’s exact test at the 50% survival rate on the day of the shortest-living condition.

### Aldicarb treatment

To treat the arrested L1 larvae with aldicarb, we allowed the eggs to hatch inside the microfluidic chambers and, after 48 hours, pipetted a 10 μL drop of aldicarb (5 mM) dissolved in water onto the agarose pad. We waited 2 hours to allow the drug to diffuse into the agarose and for the worms to absorb it. After this period, we imaged the aldicarb-treated worms as previously described. Since the total volume of agar is approximately 600 μl, the final concentration of aldicarb should be around 0.08 mM. This is much lower than the concentrations typically used to paralyze *C. elegans*, which range from 0.25 to 1.5 mM.^36^ We verified that the application of aldicarb did not cause paralysis by measuring movement speed (Figure S3L).

### Heat shock for *flp-11* overexpression

To induce the overexpression of *flp-11*-driven by the *hsp-16*.*2* promoter while minimizing stress-induced sleep, we applied a mild heat shock. We allowed the eggs to hatch inside the microfluidic chambers and, after 48 hours at 20°C, incubated the worms at 32°C for 1 hour. After waiting 2 hours for FLP-11 to be expressed, we performed DIC imaging as previously described.

### *Xenopus laevis* methods

### Plasmids

We amplified the cDNA sequence encoding *dmsr-1* by PCR using Q5 High-Fidelity DNA Polymerase (New England Biolabs (NEB), Ipswich, MA, USA) from cDNA of mixed-stage populations of wild-type *C. elegans* and cloned it into a KSM vector containing *Xenopus laevis* β-globin UTR regions and a T3 promoter, using HiFi assembly (NEB). We prepared the plasmid for the mouse M2 receptor control similarly from mouse cDNA.^27^

### Two-electrode voltage clamp (TEVC) recording

We synthesized 5′-capped cRNA using the T3 mMessage mMachine transcription kit (Thermo Fisher Scientific, Waltham, MA, USA) with KSM plasmids linearized with NotI as templates. We purified the RNA using the GeneJET RNA purification kit (Thermo Fisher Scientific). We placed *Xenopus laevis* oocytes individually into V-bottom 96-well plates and injected them with RNA using the Roboinject system (Multi Channel Systems GmbH, Reutlingen, Germany). We injected each oocyte with 50 nL of RNA mix, containing the GPCR at a final concentration of 250 ng/μL, and mGIRK1 and mGIRK2 at 150 ng/μL each. After injection, we incubated the oocytes for 2 days in ND96 at 16°C until recording.

We performed TEVC recordings using the Robocyte2 recording system (Multi Channel Systems) using recording electrodes (Multi Channel Systems) with a resistance of 0.7–2 MΩ. The probe pipettes were filled with electrode solution (1.5 M KCl and 1 M acetic acid) prior to their use. On the day of the recording, we injected some oocytes with 50 pg of pertussis toxin (Thermo Fisher Scientific) and incubated them at room temperature for 7h. We clamped the oocytes at −80 mV and continuously recorded the current at 500 Hz while flushing with ND96 for 20s, high K+ solution (96 mM KCl, 1 mM MgCl2, 5 mM HEPES, 1.8 mM CaCl2, 2 mM NaCl) for 20s, agonist solutions at 100 nM in high K^+^ for 30s, and then rinsed with ND96. The two agonists, FLP-11-3 (NGAPQPFVRFamide) and acetylcholine, were initially dissolved in DMSO at a concentration of 5 mM. We used M2, a known G_i/o_-coupled GPCR, as a positive control.^13,27^

## Quantification and Statistical Analysis

### Sleep detection based on quantification of movement quiescence

We defined sleep as periods of motion quiescence.^56–59^ We detected motion quiescence either by using differential interference contrast (DIC) imaging combined with frame subtraction or by tracking the positions of fluorescently labeled neurons during calcium imaging.^18,33,60^

### Motion detection using DIC imaging

In differential interference contrast (DIC) images, we measured worm mobility by subtracting the image intensity between two subsequent frames, which provided a measure of motion-induced changes. From the raw DIC images containing multiple animals in microwells, we cropped regions of interest (ROIs) to isolate individual worms for separate analysis. We used a custom-written Python script to subtract the pixel intensity matrix of one frame from that of the subsequent frame. We then averaged the values of the resulting matrix to represent the relative movement of the worm, with higher values indicating more movement.

### Motion detection using calcium imaging data

In the calcium imaging data, we determined the movement of the animal by tracking the head neurons (e.g., RIS) as a proxy. First, we calculated the center of mass of the pixels corresponding to the neuron expressing the fluorescent sensor. Next, we calculated the Euclidean distance of the center of mass between positions in two consecutive image frames. We converted the pixel value of the distance to micrometers using the microscope’s calibration. Finally, we calculated the movement speed from the frame rate.

### Scoring of sleep from motion data

We detected sleep from the motion datasets obtained from either frame subtraction or neuron tracking. In 0.03% of the frames, we could not calculate motion speed due to either failed detection of the neuron or because it was the first time point imaged. To address these missing values, we linearly interpolated the speed values for each worm. We then smoothed the speed values by applying the Savitzky-Golay filter with a window of 40 values, normalizing them between 0 and 1. To identify movement quiescence, we used an empirically defined speed threshold, set between 0.1 and 0.3. We scored speed values below this threshold as quiescence (assigning them a score of 0), while values above the threshold were scored as movement (assigned a score of 1). We scored sleep from these binary quiescence-movement traces through two steps: 1) We defined sleep as a period of movement quiescence lasting at least 2 minutes, while periods of quiescence shorter than 2 minutes, along with motion periods, were scored as wakefulness. 2) Brief movements (twitching) during sleep were scored as a brief wake phase, leading to the impression of fragmented sleep. To specifically remove these brief twitching periods from the score, we tested whether quiescence occurred in the two minutes before the putative bout start or after the sleep bout ended. If the worm was scored as quiescent at one or more time points within those two minutes, we calculated the average speed of movement during that period. If the average speed exceeded the speed threshold, we scored the worm as awake; if it was lower, we scored the time period as sleep. We repeated this procedure until we detected no changes in the start or end positions of sleep bouts.

### Functional Ca^2+^ imaging analysis

We measured neuronal activity using calcium imaging in animals expressing GCaMP in specific neurons of interest. We extracted the intensity of the pixels corresponding to a single neuron (e.g., RIS) as specified below.

### RIS intensity extraction

We first created a mask corresponding to the RIS cell body to extract the fluorescent signal intensities. To generate this mask from the mKate2 and GCaMP3 images, we reduced noise by applying a Gaussian blur. We then calculated a threshold using Yen’s method^61^ to create a mask encircling high-intensity pixels in the blurred image. From this mask, we retrieved the contours and calculated the area they encircled, selecting the contour with the largest area, which typically corresponded to RIS (with less than 1 in 1000 frames failing to detect RIS). We applied the RIS contour calculated from the mKate2-blurred image as a mask to select the pixels corresponding to both the mKate2 and GCaMP signals in the original non-blurred image, ensuring that their size and shape remained identical. The position of RIS occasionally shifted between the corresponding mKate2 and GCaMP3 images due to worm movement during the acquisition. To correct for this, we determined the movement of the centroid positions of RIS from the blurred GCaMP3 and mKate2 images. We then adjusted the position of the mKate2 mask based on this displacement information to extract the GCaMP3 RIS signal. To obtain a single value representing RIS signal intensity, we averaged the pixel intensities within the RIS contour for both mKate2 and GCaMP3 images. Finally, we calculated the ratio of GCaMP to mKate2 to correct for expression and focus variability.

### Sleep onset alignment

To study RIS activity at sleep onset, we extracted all sleep bouts and aligned them to their onset, defined as time point 0, when the sleep bout begins. We used data starting 15 minutes prior to and extending 25 minutes after the start of each sleep bout for averaging. GCaMP intensity was smoothed using the Savitzky-Golay filter with a window of 20 values. We first averaged the sleep score and the RIS GCaMP intensities for each individual worm, where each worm represented an N of one, and then averaged the values across different worms. For statistical testing, we averaged the GCaMP values for each individual animal within specific time intervals. We then conducted a Wilcoxon rank-sum test on the averaged values between the mutant and its controls. For the relevant intervals, we chose the time range from -10 minutes to -5 minutes relative to the sleep bout start to compare RIS GCaMP baseline values. To compare RIS GCaMP activity during sleep, we tested the interval from the bout start to 5 minutes.

### RIS peak alignment

To study how RIS activity affects sleep behavior, we averaged all RIS calcium activation transients. We identified these transients by smoothing the GCaMP trace of each worm using the Savitzky-Golay filter with a window of 120 values. We then detected transient peaks using the SciPy function scipy.signal.find_peaks (https://github.com/scipy/scipy/blob/v1.13.1/scipy/signal/_peak_finding.py#L729-L1010), which provides the indices of the peaks along with their prominence. Only peaks with a prominence of at least 0.02 were retained. Using the positional information of all detected peaks, we calculated the number and frequency of RIS activation peaks for each animal, resulting in one n, and then averaged these values across all animals.

Smoothing with a Savitzky-Golay filter using a window of 120 values preserves the larger RIS calcium transients but results in the loss of smaller transients. To extract RIS activity and motion data, we thus applied the Savitzky-Golay filter again, but this time with a smaller window of 20 values for the raw GCaMP and speed data. Smoothing with the Savitzky-Golay filter causes a rightward shift in the curve, with this effect becoming more pronounced as the window size increases. Therefore, we corrected the positional information of the prominent peaks obtained after smoothing with a window of 120 values when using this information on a dataset smoothed with a window of 20 values. To do this, we used peak width, calculated at 50% of its height, to define the peak’s position. First, we defined an interval around the original peak position to search for the highest GCaMP3 intensity. The interval was centered on the peak’s original position and extended to the left and right by a width of the peak plus 25 additional values (empirically defined). Within this interval, we identified the highest GCaMP3 intensity in the dataset smoothed with 20 values. The position of this highest intensity was then used to replace the original peak position. Finally, we used the corrected positions to extract GCaMP3 intensity and behavioral data from the dataset, analyzing data from 15 minutes before and 25 minutes after each peak for each individual worm. The GCaMP3 and behavioral data were averaged for each individual worm, resulting in one value per worm (n). These values were then averaged across all worms. For statistical testing, we averaged the GCaMP3 and sleep score values for each worm within specific time intervals. We then performed a Wilcoxon rank-sum test on the n values between the mutant and its control strains. For statistical analysis, we selected the time interval from -1 minute to +1 minute relative to the peak to examine both the maximum RIS GCaMP3 intensity and the sleep it induced.

### Quantification of HSP-12.6::mKate2 expression

To quantify the expression of HSP-12.6, we first cropped ROIs containing individual microchambers, each with one worm, and analyzed them separately. We used Otsu’s method,^62^ which utilizes the average intensity of pixels above the threshold to separate the HSP12.6::mKate2 signal from the background. We then averaged the signal above the threshold across all individuals. Internal controls were always included to account for day-to-day variability in mKate2 intensity. We averaged the intensity values from the last four days across all worms for statistical analysis. Finally, we performed the Wilcoxon rank-sum test on the averaged values.

### Sample size and number of replicates

The optimal sample size depends on factors such as effect size and reproducibility, and it can vary between experiments. There are two main reasons to increase the number of replicates: 1) to increase the sample size (n), and 2) to assess the reproducibility of the experiment. While more replicates are generally better, excessive replication can be costly and may not always be feasible due to resource constraints.

We reasoned that if an experiment is repeated twice, and if the total sample size is large enough to achieve statistical significance, and both replicates yield the same result, the experiment can be considered reproducible within the lab. If the results differ or the overall n is not sufficient, additional replicates are necessary.

Additionally, we typically included internal controls (e.g., wild-type backgrounds) to account for day-to-day variation. This allowed us to quickly detect any general problems with the measurements. Data for individual replicates can be found in the supplement (Data S4).

## Supplementary Material

Supplemental information can be found online at https://doi.org/10.1016/j.cub.2025.03.039.

A video abstract is available at https://doi.org/10.1016/j.cub.2025.03.039#mmc6.

Data S1. Sequence comparison of dmsr-1 alleles, related to Figures 1, 2, 3, and S1–S3.

Data S2.

Data S3.

Data S4.

Document S1. Figures S1–S4.

## Data Availability

Raw and processed data for all experiments, as well as Python scripts used for analysis, can be accessed at the DOIs that are listed in the key resources table. All of the original data are available publicly as indicated above. Any additional information required to reanalyze the data reported in this paper is available from the lead contact upon request.

## Highlights

- DMSR-1 regulates sleep downstream of RIS and FLP-11 RFamide neuropeptides
- DMSR-1 is an inhibitory GPCR because it directly couples with G_i/o_ protein
- DMSR-1 is required in cholinergic neurons for sleep induction downstream of RIS
- DMSR-1 is necessary in the sleep-active RIS neuron for limiting sleep duration

## In brief

Rossi et al. show that FLP-11 neuropeptides, released from the *C. elegans* sleep neuron RIS, induce sleep by inhibiting cholinergic neurons through the G_i/o_-coupled receptor DMSR-1. Because RIS also expresses DMSR-1, it inhibits its own activity upon activation. This mechanism effectively links sleep induction to the limitation of sleep duration.
