# Supplementary material for: The neuropeptide FLP-11 induces and self-inhibits sleep through the receptor DMSR-1 in Caenorhabditis elegans
Source: Curr Biol. Author manuscript; Available in PMC 2025 Jun 24. (PMC7617803; doi:10.1016/j.cub.2025.03.039)
Supplement: Data S1. Sequence comparison of dmsr-1 alleles, related to Figures 1, 2, 3, and S1–S3. [file EMS206526-supplement-Data_S1__Sequence_comparison_of_dmsr_1_alleles__related_to_Figures_1__2__3__and_S1_S3_.pdf]

dmsr-1 deletion allele sequences

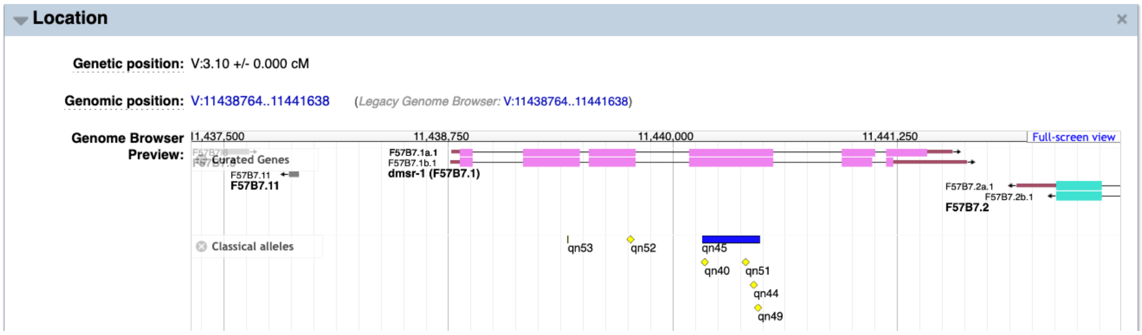

TMHMM result

```
# WEBSEQUENCE Length: 510
# WEBSEQUENCE Number of predicted TMHs:  7
# WEBSEQUENCE Exp number of AAs in TMHs: 150.23496
# WEBSEQUENCE Exp number, first 60 AAs:  22.79728
# WEBSEQUENCE Total prob of N-in:        0.00310
# WEBSEQUENCE POSSIBLE N-term signal sequence
WEBSEQUENCE	TMHMM2.0	outside	     1    32
WEBSEQUENCE	TMHMM2.0	TMhelix	    33    55
WEBSEQUENCE	TMHMM2.0	inside	    56    67
WEBSEQUENCE	TMHMM2.0	TMhelix	    68    90
WEBSEQUENCE	TMHMM2.0	outside	    91   109
WEBSEQUENCE	TMHMM2.0	TMhelix	   110   132
WEBSEQUENCE	TMHMM2.0	inside	   133   152
WEBSEQUENCE	TMHMM2.0	TMhelix	   153   175
WEBSEQUENCE	TMHMM2.0	outside	   176   222
WEBSEQUENCE	TMHMM2.0	TMhelix	   223   245
WEBSEQUENCE	TMHMM2.0	inside	   246   306
WEBSEQUENCE	TMHMM2.0	TMhelix	   307   329
WEBSEQUENCE	TMHMM2.0	outside	   330   343
WEBSEQUENCE	TMHMM2.0	TMhelix	   344   366
WEBSEQUENCE	TMHMM2.0	inside	   367   510
```

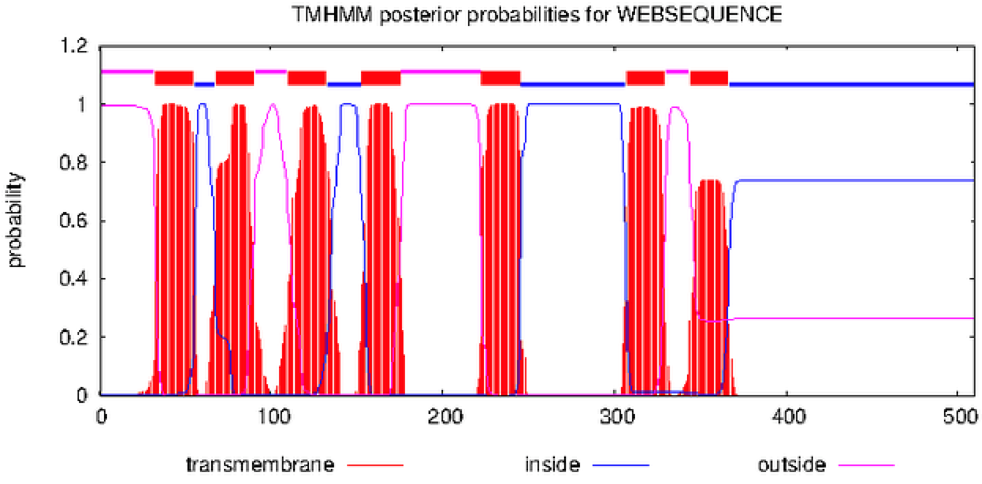

> dmsr-1 Unspliced coding + 7TM
ATGGAGTTTACCGAATGCAAAACTACATTTATTCATCTGCCCGATAAAAGTTTTTTATA
CGATGTTTTTGTgtaagttttacttttgtgtctcaaaaatctttcaataattgcccag
tactgttgtatttgacgtattttcaaaaagtacagtttatcatcattaaaagttatcct
attatttgtagaaatcatccctaaaaatagcgaataaaaccaagttattgaagaaggtt

atcttatcttaaattattgtgtcctcaaaagtgatgaccataaaaaagagagccattaa
taaatatttcaagagcaaaagtggaaaaaagtgaaattaaacgtttttttttcagAAGT
GTATATAATTTCTACCATCCTATACATGCCTACTTATCAATATTTCTATGCGTGTTGGG
TACAATCGCTAATTTCTGTAACATCGTCGTACTAACGAGACGAACAATGCGAACGCCAG
TTAATATGATTTTGACAGCAATGGCATCTTGTGATACAGTTGTGTTATTTTCAAATTTA
ATATACACAACACATTACTCATTTGTCGCTTTCAAGTTTTGTCATCCGAAACATTGGTC
CTACTCTTGGGCGTTATTTTTAATTGCTCATGCTCATCTTTCATTAGTTGCACATTCTT
CAAGTGTTTGGTTGTCAGgtaagcttttcattctgaagacaactgtccaactcttcgat
tttattgcagTTATGCTTGCACTTGTTCGATATGTAACACTTCGAAGTAGGGGAAATAT
GGGTGGTATGCAAGTGACGTTAAGGCATTCTTATTATGCCGTTGCTGTTACTGTATCCC
TTGTAGCAGTGCTTAATGCACCGAATTTTCTGAATTACAAAATCAATGAACAGCCATTG
AATGAAACGTGTACCGATTTGGATCCAATGTTCTGGAATTCGCCCGCGTATCTTCCTGG
AATTGCAGACATTGCAAAAGCAAACAGCTGTCTGgtaagattttgaatttcagtaaaa
aaaaaacaagattttgttaaaaaataatgtttgacaaattgtatacaaacaaaatttc
tcgaaaaactttttattggaacttagatttataaactaaaaatttggcaagatcatc
ataatataactttgaagctttggatttatataaacctttttggaaataattgatcgatc
atatgtacatatgatatcatgaaaattatacaaaataatatttatcatcgtgatcgtca
ccctagcaaaaaccaatttcctaatttcctgcagGTCTTCCGGTTATCTTATTGGATAT
CCGGTATGGTATTCAAAGTATTACCATGTGCGCTTCTATCGTTGTTTGTTTGGCTCCTT
TTACGAATTCTTCGTGAAGTGCGTGAGAATCGTCAACGTCTTCTCAAGAACTCGCAACA
TCGACCACCGAATCAAACGACTACTCGAAACGGACAAAGACTGAGCATTTCAGTTGCAG
GCAACGAGAAATTAGGCAGAAATGGAAGCTTACGGGGGAGAGGAGAACGTGTCGATCGG
ACAACTCATATGTTATTGGCAATTGTAGCAGTTATGCTAGTGACTGAATTACCTCAAGG
AATTATGGCTGTCTTGTCTGGAATGTGTTCTGAAGAATTCCGAATTTACATTTATAACA
ATCTGGGAGACATTCTCGATTTGTTCTCACTTTGCGGTTCATGTTGTTCATTCATCATT
TACTGCTCAATGAGTGGACAGTTCAGAAATgtaagttttgatattacttgaaaaaaatt
tcaatgagcgacatcttttcaaaatacctcaatgaaaaatgtgttgtatgctgttagaa
aatactacaaactttctcaacattcggtggaactttccaagcgaatttgtcttttttttt
ctttgttttcttactaacatgatgttcggcagcgtcttaaaatgcgcttaagatctcta
cattttccgacaattgcggagttgtttttacaagaaaaatcatggaagtacatactttg
ctcattttatgaatgtgtgtttagttcattttaacacagggaacaagcattttcatttg
atttctataaataaaaattggttcattttcaatatagaaaaatcataaaatttttagGA
ATTCCACCGTGTCTTTGTACCTGCAAAGGTGAGATGTCTCCGAATGTCATCGCCGTCGA
TTCGTCGTCCATCCGACGCCTACAGTACCACAAAAATGACTTTTCTAAAACCAAACGAG
AAAAACGGAAATGGAATGAATGGAAATGGCACTTATTCGGAAGATACAAGGTCAGCAAG
TGTTAAAgtaagaatatcaacacatgtttttttctacaaaacttttgaataagccatgtt
aattttcagATGGTCGGAATCCAGGTACGAAGAAACAGTACGGAAATAACGAGAATGAC
TGGATGTGATTCAATTACTCCATGTTCTCCAATGCCAACATCATTTCCATCATCCCCAC
TTCCACCGATTCGAAGTGGAGAAGATGAATCCACTGATGAGACATCACATCTACTTAAC
AGCTCAGGACCCAACTCAACAGCCAGTGCTGATGGAATTCGTGGACACTTTCAAAACAT
TTGA

>dmsr-1(syb6331syb6333[FRT::dmsr-1 exons 2-3::FRT]) V.
unspliced coding + 7TM
ATGGAGTTTACCGAATGCAAAACTACATTTATTCATCTGCCCGATAAAAGTTTTTTATA
CGATGTTTTTGTgtaagttttacttttgtgtctcaaaaatctttcaaataattgcccag

tactgttgtatttgacgtattttcaaaaagtacagtttatcatcattaaaagttatcct
attatttgtagaaatcatccctaaaaatagcgaataaaaccaagttattgaagaaggtt
atcttatcttaaattattgtgtcctcaaaagtgatgaccataaaaaagagagccattaa
taaatatttcaagagcaaaagtggGAAGTTCCTATTCtctagaaaGtATAGGAACTTCa
aaaaagtgaaattaaacgtttttttttcagAAGTGTATATAATTTCTACCATCCTATAC
ATGCCTACTTATCAATATTTCTATGCGTGTTGGGTACAATCGCTAATTTCTGTAACATC
GTCGTACTAACGAGACGAACAATGCGAACGCCAGTTAATATGATTTTGACAGCAATGGC
ATCTTGTGATACAGTTGTGTTATTTTCAAATTTAATATACACAACACATTACTCATTTG
TCGCTTTCAAGTTTTGTCATCCGAAACATTGGTCCTACTCTTGGGCGTTATTTTTAATT
GCTCATGCTCATCTTTCATTAGTTGCACATTCTTCAAGTGTTTGGTTGTCAGgtaagct
tttcattctgaagacaactgtccaactcttcgattttattgcagTTATGCTTGCACTTG
TTCGATATGTAACACTTCGAAGTAGGGGAAATATGGGTGGTATGCAAGTGACGTTAAGG
CATTCTTATTATGCCGTTGCTGTTACTGTATCCCTTGTAGCAGTGCTTAATGCACCGAA
TTTTCTGAATTACAAAATCAATGAACAGCCATTGAATGAAACGTGTACCGATTTGGATC
CAATGTTCTGGAATTCGCCCGCGTATCTTCCTGGAATTGCAGACATTGCAAAAGCAAAC
AGCTGTCTGgtaagattttgaatttcagtaaaaaaaaaacaaGAAGTTCCTATTCtct
agaaaGtATAGGAACTTCgatttttgttaaaaaataatgtttgacaaattgtatacaaa
caaaatttctcgaaaaactttttattggaacttagatttataaactaaaaattttggc
aagatcatcataatataactttgaagctttggatttatataaacctttttggaaataat
tgatcgatcatatgtacatatgatatcatgaaaattatacaaaataatatttatcatcg
tgatcgtcaccctagcaaaaaccaatttcctaatttcctgcagGTCTTCCGGTTATCTT
ATTGGATATCCGGTATGGTATTCAAAGTATTACCATGTGCGCTTCTATCGTTGTTTGTT
TGGCTCCTTTTACGAATTCTTCGTGAAGTGCGTGAGAATCGTCAACGTCTTCTCAAGAA
CTCGCAACATCGACCACCGAATCAAACGACTACTCGAAACGGACAAAGACTGAGCATTT
CAGTTGCAGGCAACGAGAAATTAGGCAGAAATGGAAGCTTACGGGGGAGAGGAGAACGT
GTCGATCGGACAACTCATATGTTATTGGCAATTGTAGCAGTTATGCTAGTGACTGAATT
ACCTCAAGGAATTATGGCTGTCTTGTCTGGAATGTGTTCTGAAGAATTCCGAATTTACA
TTTATAACAATCTGGGAGACATTCTCGATTTGTTCTCACTTTGCGGTTCATGTTGTTCA
TTCATCATTTACTGCTCAATGAGTGGACAGTTCAGAAATgtaagttttgatattacttg
aaaaaaatttcaatgagcgacatcttttcaaaatacctcaatgaaaaatgtgttgtatg
ctgttagaaaatactacaaactttctcaacattcggtggaactttccaagcgaatttgt
ctttttttctttgttttcttactaacatgatgttcggcagcgtcttaaaatgcgctta
agatctctacattttccgacaattgcggagttgtttttacaagaaaaatcatggaagta
catactttgctcattttatgaatgtgtgtttagttcattttaacacagggaacaagcat
tttcatttgatttctataaataaaaattggttcattttcaatatagaaaaatcataaaa
tttttagGAATTCCACCGTGTCTTTGTACCTGCAAAGGTGAGATGTCTCCGAATGTCAT
CGCCGTCGATTCGTCGTCCATCCGACGCCTACAGTACCACAAAAATGACTTTTCTAAAA
CCAAACGAGAAAAACGGAAATGGAATGAATGGAAATGGCACTTATTCGGAAGATACAAG
GTCAGCAAGTGTTAAAgtaagaatatcaacacatgtttttctacaaaacttttgaata
agccatgttaattttcagATGGTCGGAATCCAGGTACGAAGAAACAGTACGGAAATAAC
GAGAATGACTGGATGTGATTCAATTACTCCATGTTCTCCAATGCCAACATCATTTCCAT
CATCCCCACTTCCACCGATTCGAAGTGGAGAAGATGAATCCACTGATGAGACATCACAT
CTACTTAACAGCTCAGGACCCAACTCAACAGCCAGTGCTGATGGAATTCGTGGACACTT
TCAAAACATTTGA

>dmsr-1(syb6331syb6333[FRT]) V. (deletes the first four TMs and introduces a frame shift)
ATGGAGTTTACCGAATGCAAAACTACATTTATTCATCTGCCCGATAAAAGTTTTTATA
CGATGTTTTTGTgtaagttttactttgtgtctcaaaaatctttcaaataattgcccag
tactgttgtatttgacgtattttcaaaaagtacagtttatcatcattaaaagttatcct
attatttgtagaaatcatccctaaaaatagcgaataaaaccaagttattgaagaaggtt
atcttatcttaaattattgtgtcctcaaaagtgatgaccataaaaaagagagccattaa
taaatatttcaagagcaaaagtggGAAGTTCCTATTCtctagaaaGtATAGGAACTTCg
atttttgttaaaaaataatgtttgacaaattgtatacaaacaaaatttctcgaaaaact
ttttattggaacttagatttataaactaaaaatttttggcaagatcatcataatataac
tttgaagctttggatttatataaaccttttttggaaataattgatcgatcatatgtacat
atgatatcatgaaaattatacaaaataatatttatcatcgtgatcgtcaccctagcaaa
aaccaatttcctaatttcctgcagGTCTTCCGGTTATCTTATTGGATATCCGGTATGGT
ATTCAAAGTATTACCATGTGCGCTTCTATCGTTGTTTGTTTGGCTCCTTTTACGAATTC
TTCGTGAAGTGCGTGAGAATCGTCAACGTCTTCTCAAGAACTCGCAACATCGACCACCG
AATCAAACGACTACTCGAAACGGACAAAGACTGAGCATTTCAGTTGCAGGCAACGAGAA
ATTAGGCAGAAATGGAAGCTTACGGGGGAGAGGAGAACGTGTCGATCGGACAACTCATA
TGTTATTGGCAATTGTAGCAGTTATGCTAGTGACTGAATTACCTCAAGGAATTATGGCT
GTCTTGTCTGGAATGTGTTCTGAAGAATTCCGAATTTACATTTATAACAATCTGGGAGA
CATTCTCGATTTGTTCTCACTTTGCGGTTCATGTTGTTCATTCATCATTTACTGCTCAA
TGAGTGGACAGTTCAGAAATgtaagttttgatattacttgaaaaaaatttcaatgagcg
acatcttttcaaaatacctcaatgaaaaatgtgttgtatgctgttagaaaatactacaa
actttctcaacattcggtggaactttccaagcgaatttgtctttttttttctttgttttc
ttactaacatgatgttcggcagcgtcttaaaatgcgcttaagatctctacattttccga
caattgcggagttgtttttacaagaaaaatcatggaagtacatactttgctcattttat
gaatgtgtgtttagttcattttaacacagggaacaagcattttcatttgatttctataa
ataaaaattggttcattttcaatatagaaaaatcataaaatttttagGAATTCCACCGT
GTCTTTGTACCTGCAAAGGTGAGATGTCTCCGAATGTCATCGCCGTCGATTCGTCGTCC
ATCCGACGCCTACAGTACCACAAAAATGACTTTTCTAAAACCAAACGAGAAAAACGGAA
ATGGAATGAATGGAAATGGCACTTATTCGGAAGATACAAGGTCAGCAAGTGTTAAAgta
agaatatcaacacatgtttttttctacaaaacttttgaataagccatgttaattttcagA
TGGTCGGAATCCAGGTACGAAGAAACAGTACGGAAATAACGAGAATGACTGGATGTGAT
TCAATTACTCCATGTTCTCCAATGCCAACATCATTTCCATCATCCCCACTTCCACCGAT
TCGAAGTGGAGAAGATGAATCCACTGATGAGACATCACATCTACTTAACAGCTCAGGAC
CCAACTCAACAGCCAGTGCTGATGGAATTCGTGGACACTTTCAAAACATTTGA

>dmsr-1(pq45) deletion
ATGGAGTTTACCGAATGCAAAACTACATTTATTCATCTGCCCGATAAAAGTTTTTATA
CGATGTTTTTGTgtaagttttactttgtgtctcaaaaatctttcaaataattgcccag
tactgttgtatttgacgtattttcaaaaagtacagtttatcatcattaaaagttatcct
attatttgtagaaatcatccctaaaaatagcgaataaaaccaagttattgaagaaggtt
atcttatcttaaattattgtgtcctcaaaagtgatgaccataaaaaagagagccattaa
taaatatttcaagagcaaaagtggaaaaaagtgaaattaaacgtttttttttcagAAGT
GTATATAATTTCTACCATCCTATACATGCCTACTTATCAATATTTCTATGCGTGTTGGG
TACAATCGCTAATTTCTGTAACATCGTCGTACTAACGAGACGAACAATGCGAACGCCAG
TTAATATGATTTTGACAGCAATGGCATCTTGTGATACAGTTGTGTTATTTTCAAATTTA
ATATACACAACACATTACTCATTTGTCGCTTTCAAGTTTTGTCATCCGAAACATTGGTC

CTACTCTTGGGCGTTATTTTTAATTGCTCATGCTCATCTTTCATTAGTTGCACATTCTT
CAAGTGTTTGGTTGTCAGgtaagcttttcattctgaagacaactgtccaactcttcgat
tttattgcagTTATGCTTGCACTTGTTCGATATGTAACACTTCGAAGTAGGGGAAATAT
GGGTGGTATGCAAGTGACGTTAAGGCATTCTTATTATGCCGTTGCTGTTACTGTATCCC
TTGTAGCAGTGCTTAATGCACCGAATTTTCTGAATTACAAAATCAATGAACAGCCATTG
AATGAAACGTGTACCGATTTGGATCCAATGTTCTGGAATTCGCCCGCGTATCTTCCTGG
AATTGCAGACATTGCAAAAGCAAACAGCTGTCTGgtaagattttgaatttcagtaaaa
aaaaaacaagattttgttaaaaaataatgtttgacaaattgtatacaaacaaaatttc
tcgaaaaactttttattggaacttagatttataaactaaaaatttggcaagatcatc
ataatataactttgaagctttggatttatataaaccttttggaaataattgatcgatc
atatgtacatatgatatcatgaaaattatacaaaataatatttatcatcgtgatcgtca
ccctagcaaaaaccaatttcctaatttcctgcagGTCTTCCGGTTATCTTATTGGATAT
CCGGTATGGTATTCAAAGTATTACCATGTGCGCTTCTATCGTTGTTT

-

TCGATTTGTTCTCACTTTGCGGTTCATGTTGTTCATTCATCATTTACTGCTCAATGAGT
GGACAGTTCAGAAATgtaagttttgatattacttgaaaaaaatttcaatgagcgacatc
ttttcaaaatacctcaatgaaaaatgtgttgtatgctgttagaaaatactacaaacttt
ctcaacattcggtggaactttccaagcgaatttgtctttttttttctttgttttcttact
aacatgatgttcggcagcgtcttaaaatgcgcttaagatctctacattttccgacaatt
gcggagttgtttttacaagaaaaatcatggaagtacatactttgctcattttatgaatg
tgtgtttagttcattttaacacagggaacaagcattttcatttgatttctataaataaa
aattggttcattttcaatatagaaaaatcataaaatttttagGAATTCCACCGTGTCTT
TGTACCTGCAAAGGTGAGATGTCTCCGAATGTCATCGCCGTCGATTCGTCGTCCATCCG
ACGCCTACAGTACCACAAAAATGACTTTTCTAAAACCAAACGAGAAAAACGGAAATGGA
ATGAATGGAAATGGCACTTATTCGGAAGATACAAGGTCAGCAAGTGTTAAAgtaagaat
atcaacacatgtttttctacaaacttttgaataagccatgttaattttcagATGGTC
GGAATCCAGGTACGAAGAAACAGTACGGAAATAACGAGAATGACTGGATGTGATTCAAT
TACTCCATGTTCTCCAATGCCAACATCATTTCCATCATCCCCACTTCCACCGATTCGAA
GTGGAGAAGATGAATCCACTGATGAGACATCACATCTACTTAACAGCTCAGGACCCAAC
TCAACAGCCAGTGCTGATGGAATTCGTGGACACTTTCAAAACATTTGA

>*dmsr-1(sy1522)* V.
ATGGAGTTTACCGAATGCAAAACTACATTTATTCATCTGCCCGATAAAAGTTTTTTATA
CGATGTTTTTGTgtaagttttacttttgtgtctcaaaaatctttcaaataattgcccag
tactgttgtatttgacgtattttcaaaaagtacagtttatcatcattaaaagttatcct
attatttgtagaaatcatccctaaaaatagcgaataaaaccaagttattgaagaaggtt
atcttatcttaaattattgtgtcctcaaaagtgatgaccataaaaaagagagccattaa
taaatatttcaagagcaaaagtggaaaaaagtgaaattaaacgtttttttttcagAAGT
GTATATAATTTCTACCATCCTATACATGCCTACTTATCAATATTTCTATGCGT
GGGAAGTTTGTCCAGAGCAGAGGTGACTAAG**TGATAA**gctagc
GTTGGGTACAATCGCTAATTTCTGTAACATCGTCGTACTAACGAGACGAACAATGCGAA
CGCCAGTTAATATGATTTTGACAGCAATGGCATCTTGTGATACAGTTGTGTTATTTTCA
AATTTAATATACACAACACATTACTCATTTGTCGCTTTCAAGTTTTGTCATCCGAAACA
TTGGTCCTACTCTTGGGCGTTATTTTTAATTGCTCATGCTCATCTTTCATTAGTTGCAC
ATTCTTCAAGTGTTTGGTTGTCAGgtaagcttttcattctgaagacaactgtccaactc
ttcgattttattgcagTTATGCTTGCACTTGTTCGATATGTAACACTTCGAAGTAGGGG

AAATATGGGTGGTATGCAAGTGACGTTAAGGCATTCTTATTATGCCGTTGCTGTTACTG
TATCCCTTGTAGCAGTGCTTAATGCACCGAATTTTCTGAATTACAAAATCAATGAACAG
CCATTGAATGAAACGTGTACCGATTTGGATCCAATGTTCTGGAATTCGCCCGCGTATCT
TCCTGGAATTGCAGACATTGCAAAAGCAAACAGCTGTCTGgtaagattttgaatttca
gtaaaaaaaaaacaagattttgttaaaaaataatgtttgacaaattgtatacaaacaa
aatttctcgaaaaactttttattggaacttagatttataaactaaaaattttggcaag
atcatcataatataactttgaagctttggatttatataaaccttttggaaataattga
tcgatcatatgtacatatgatatcatgaaaattatacaaaataatatttatcatcgtga
tcgtcaccctagcaaaaaccaatttcctaatttcctgcagGTCTTCCGGTTATCTTATT
GGATATCCGGTATGGTATTCAAAGTATTACCATGTGCGCTTCTATCGTTGTTTGTTTGG
CTCCTTTTACGAATTCTTCGTGAAGTGCGTGAGAATCGTCAACGTCTTCTCAAGAACTC
GCAACATCGACCACCGAATCAAACGACTACTCGAAACGGACAAAGACTGAGCATTTCAG
TTGCAGGCAACGAGAAATTAGGCAGAAATGGAAGCTTACGGGGGAGAGGAGAACGTGTC
GATCGGACAACTCATATGTTATTGGCAATTGTAGCAGTTATGCTAGTGACTGAATTACC
TCAAGGAATTATGGCTGTCTTGTCTGGAATGTGTTCTGAAGAATTCCGAATTTACATTT
ATAACAATCTGGGAGACATTCTCGATTTGTTCTCACTTTGCGGTTCATGTTGTTCATTC
ATCATTTACTGCTCAATGAGTGGACAGTTCAGAAATgtaagttttgatattacttgaaa
aaaatttcaatgagcgacatcttttcaaaatacctcaatgaaaaatgtgttgtatgctg
ttagaaaatactacaaactttctcaacattcggtggaactttccaagcgaatttgtctt
tttttctttgttttcttactaacatgatgttcggcagcgtcttaaaatgcgcttaaga
tctctacattttccgacaattgcggagttgtttttacaagaaaaatcatggaagtacat
actttgctcattttatgaatgtgtgtttagttcattttaacacagggaacaagcatttt
catttgatttctataaataaaaattggttcattttcaatatagaaaaatcataaaattt
ttagGAATTCCACCGTGTCTTTGTACCTGCAAAGGTGAGATGTCTCCGAATGTCATCGC
CGTCGATTCGTCGTCCATCCGACGCCTACAGTACCACAAAAATGACTTTTCTAAAACCA
AACGAGAAAAACGGAAATGGAATGAATGGAAATGGCACTTATTCGGAAGATACAAGGTC
AGCAAGTGTTAAAgtaagaatatcaacacatgtttttttctacaaaacttttgaataagc
catgttaattttcagATGGTCGGAATCCAGGTACGAAGAAACAGTACGGAAATAACGAG
AATGACTGGATGTGATTCAATTACTCCATGTTCTCCAATGCCAACATCATTTCCATCAT
CCCCACTTCCACCGATTCGAAGTGGAGAAGATGAATCCACTGATGAGACATCACATCTA
CTTAACAGCTCAGGACCCAACTCAACAGCCAGTGCTGATGGAATTCGTGGACACTTTCA
AAACATTTGA
