# Supplementary material for: The neuropeptide FLP-11 induces and self-inhibits sleep through the receptor DMSR-1 in Caenorhabditis elegans
Source: Curr Biol. Author manuscript; Available in PMC 2025 Jun 24. (PMC7617803; doi:10.1016/j.cub.2025.03.039)
Supplement: Data S3. [file EMS206526-supplement-Data_S3_.pdf]

PHX2457

>dmsr-7(syb2457)

cctgctatagaaataacaactcacggaacaaaaataacttccggccagtgacctataatttt
gtaaatttttgaaaaacgaaattatatattttgttcgaaaactcgatcagcttcttccaca
acaaccgactgtcatctaaaagttcaaaaaacacagaacgcattccaccctgttgctcttgg
cttcttctgattttctccttatgtttctttgtttcaatggtctttaaaaccccttaaattt
tcaacattttgaaggatgtgctagat-
attccaatttttcgatctcggtacaaattccctttgcagatttttgtgatatcttgctca
ttttcatagctttcgcattttctattctattcttgttacgggtactttttgtttttattaa
ttcagttttatataaatcaatatggtttacaaaattattaaattattcacggcgccgtgaa
aaaaaaattgtttacaaaaagacacgtagatgtgagtaaaactaggaaaaaaaattattttc
aattgaaagtgatttccgaaaggtgagttctctttttctcttccaggaatcaaaagttaaaa
aggtttgatgttttagattatgaaagacgacgtcgttgacaacataataattgatgtcctga
gcgctagtactc

-:deletion site

PHX5866

>flp-11(syb5866)

cacaagtaggcgtggcctgtggaacgtttcagagcgcagaacacctgcatttgatctattca
cttcttgcttttgaaaagcccaaagacaccctacacttcggtttcgttttggaaaccattga
catcatcctattttccataagaagtttccttgagaagaatccatttcgcaaatttttcatta
aaacgttcaaaactcatcaaaccatttgtaaatagtaataaagtatgtcctgcggctatttg
ctttctcttcggaatctacaacgccccctcctaatacatcgtttcaggtataaaaagactgc
gcct**ag**g-
acactctctttttttcatactctctcttgctgtctagaatttgattggtgtcgcttaacccccc
ctttccctccgaaggaaagttatctccccagatctcttttggtgtttttttatcagctaacaa
cacacattttctgatatttctatgctctgtctatgaacaataaaggcgttgttaattactcg
caaaatcactttgtttatttttttcacattttcagatagtgaacaaaagaaaattaaattct
aaaatctgaatcggaaaattcaaattaaaaattaaatttatttttttttatattacacctgtt
ttttttcaaatattagatcaaaaactattcaacaagtggcatgtaaagcata

-:deletion site

PHX6333

Synonymous mutations are labeled in turquoise.

>dmsr-1(syb6331syb6333[FRT exons 2-3 FRT])

ATGGAGTTTACCGAATGCAAAACTACATTTATTCATCTGCCCGATAAAAGTTTTTTATACGA
TGTTTTTGTgtaagttttactttttgtgtctcaaaaatctttcaaataattgcccagtactgt
tgtatttgacgtattttcaaaaagtacagtttatcatcattaaaagttatcctattatttgt
agaaatcatccctaaaaatagcgaataaaaccaagttattgaagaaggttatcttatcttaa
attattgtgtcctcaaaagtgatgaccataaaaaagagagc**G**attaataaatatttcaagag
caaaagt**C**gGAAGTTCCTATTCTCTAGAAAGTATAGGAACTTCaaaaaagtgaaattaaacg
ttttttttcagAAGTGTATATAATTTC**TAT**CATCCT**ATC**CATGCCTACTTATCAATATTTC
TATGCGTGTTGGGTACAATCGCTAATTTCTGTAACATCGTCGTACTAACGAGACGAACAATG
CGAACGCCAGTTAATATGATTTTGACAGCAATGGCATCTTGTGATACAGTTGTGTTATTTTC
AAATTTAATATACACAACACATTACTCATTTGTCGCTTTCAAGTTTTGTCATCCGAAACATT
GGTCCTACTCTTGGGCGTTATTTTTAATTGCTCATGCTCATCTTTCATTAGTTGCACATTCT
TCAAGTGTTTGGTTGTCAGgtaagcttttcattctgaagacaactgtccaactcttcgattt
tattgcagTTATGCTTGCACTTGTTCGATATGTAACACTTCGAAGTAGGGGAAATATGGGTG
GTATGCAAGTGACGTTAAGGCATTCTTATTATGCCGTTGCTGTTACTGTATCCCTTGTAGCA
GTGCTTAATGCACCGAATTTTCTGAATTACAAAATCAATGAACAGCCATTGAATGAAACGTG
TACCGATTTGGATCCAATGTTCTGGAATTCGCCCGCGTAT**TTG**CCT**GGTATC**GCAGACATTG
CAAAAGCAAAC**TCC**TGTCTGgtaagattttgaatttcagtaaaaaaaaaacaaGAAGTTCC
TATTCTCTAGAAAGTATAGGAACTTCgatttttgttaaaaaataatgtttgacaaattgtat
acaaacaaaatttctcgaaaaactttttattggaacttagatttataaactaaaattttg
gcaagatcatcataatataactttgaagctttggatttatataaacctttttggaaataatt
gatcgatcatatgtacatatgatatcatgaaattatacaaaataatatttatcatcgtgat
cgtcaccctagcaaaaaccaatttcctaatttcctgcagGTCTTCCGGTTATCTTATTGGAT
ATCCGGTATGGTATTCAAAGTATTACCATGTGCGCTTCTATCGTTGTTTGTTTGGCTCCTTT
TACGAATTCTTCGTGAAGTGCGTGAGAATCGTCAACGTCTTCTCAAGAACTCGCAACATCGA
CC

PHX8025

Synonymous mutations are labeled in turquoise.

>nmr-1(syb8025[nmr-1::SL2::FLP D5])

aacaaaaagttgaacccctaaaaataagtgagtaattgttgcaaatagtttactgaaacaag
aaacgtctatacatagtttgatccaatcttaaaagtgtaacattaactttcttctaactgta
aacgagtttttccttgcaattccattcttctccttaattgttggtcactttttttgcaggcca
agtttcaaaactggacgctgaaatagaaatttatgagaccaacttttaccaataaccaataa
atttcagAACATCGTGGAATCAGATGTGCATCGCGAAACGGGTCTCTTTGCTTTTCTCTCGT
GTTTTCTGTTTGCAATTCTCTTTCTGTGGCCATGCTCTCCACTTCCTTGCTTTTTGAGCTCA
TTCAGTGATTTTGTGCACATTTGTCCATTGTGT**AGC**CATATTATGGGA**AGA**TTTCGAAGAGC
AAGATCAACTAGATTTTATGTGTGAgctgtctcatcctactttcacctagttaactgcttgt
cttaaaatctatgcttctctttagtatctaaaattttcctagaagcttacaagtatataaat
ggtctcttctcaataaaggttgtatatttattcatcttattgaatctgccatttcctcgttt
ttgcgagtttatataccttccaatttctttctattgtattttcaacttctaattttaattc
agggaaactgcttcaacgcatcATGCCACAATTCGacATTCTCTGTAAGACACCACCAAAAG
TCCTCGTCCGTCAATTCGTCGAGCGTTTCGAGCGTCCATCCGGAGAGAAGATCGCCCTCTGC
GCCGCCGAGCTCACCTACCTCTGCTGGATGATCACCCACAACGGAACCGCCATCAAGCGTGC
CACCTTCATGTCCTACAACACCATCATCTCCAACTCCCTCTCCTTCGACATCGTCAACAAGT
CCCTCCAATTCAAGTACAAGACCCAAAAGGCCACCATCCTCGAGGCCTCCCTCAAGgtaagt
ttaaacatatatatactaactaaccctgattatttaaattttcagAAGCTCATCCCAGCCTG
GGAGTTCACCATCATCCCATACTACGGACAAAAGCACCAATCCGACATCACCGACATCGTCT
CCTCCCTCCAACTCCAATTCGAGTCCTCCGAGGAGGCCGACAAGGGAAACTCCCACTCCAAG
AAGATGCTCAAGGCCCTCCTCTCCGAGGGAGAGTCCATCTGGGAGATCACCGAGAAGATCCT
CAACTCCTTCGAGTACACCTCCCGTTTCACCAAGACCAAGACCCTCTACCAATTCCTCTTCC
TCGCCACCTTCATCAACTGCGGACGTTTCTCCGACATCAAGAACGTCGACCCAAAGTCCTTC
AAGCTCGTCCAAAACAAGTACCTCGGAGTCATCATCCAATGCCTCGTCACCGAGACCAAGAC
CTCCGTCTCCCGTCACATCTACTTCTTCTCCGCCCGTGGACGTATCGACCCACTCGTCTACC
TCGACGAGTTCCTCCGTAACTCCGAGCCAGTCCTCAAGCGTGTCAACCGTACCGGAAACTCC
TCCTCCAACAAGCAAGAGTACCAACTCCTCAAGGACAACCTCGTCCGTTCCTACAACAAGGC
CCTCAAGAAGAACGCCCCATACTCCATCTTCGCCATCAAGAACGGACCAAAGTCCCACATCG
GACGTCACCTCATGACCTCCTTCCTCTCCATGAAGgtaagtttaaacatgattttactaact
aactaatctgatttaaattttcagGGACTCACCGAGCTCACCAACGTCGTCGGAAACTGGTC
CGACAAGCGTGCCTCCGCCGTCGCCCGTACCACCTACACCCACCAAATCACCGCCATCCCAG
ACCACTACTTCGCCCTCGTCTCCCGTTACTACGCCTACGACCCAATCTCCAAGGAGATGATC
GCCCTCAAGGACGAGACCAACCCAATCGAGGAGTGGCAACACATCGAGCAACTCAAGGATC
CGCCGAGGGATCCATCCGTTACCCAGCCTGGAACGGAATCATCTCCCAAGAGGTCCTCGACT
ACCTCTCCTCCTACATCAACCGTCGTATCTAAatttggcatttcatgcttctattattgcaa
tcaaattgtaaatattgtcaactcgaataaacatttaatttattttagcacaaatgagcaag
taaatgggaacggtttattcagtgtcgtgatgcttgtatagcaattttttgaaacagcaacac
cggaaaatgaaatcggcagttatcttctgaaagtagacgttttgagttcaggattccagaaa
attattggagagaagaaaagaaacggagaaatgatgcatgacgtcaattagatatcttcgta
atcgacagtcttccctttttggtcttcagtagtacaactcaacggaagaaaatagacatcag
tacagtcgtcttggaaacactgaaattatataaattgggatttatattcaacaaatatacac
ttacagccaaattgtcaatga

PHX9007

Synonymous mutation is labeled in turquoise.

>*unc-4(syb9007[unc-4::SL2::FLP D5])*

AAATGGCGAAAGCGAGAGCAAAACCGAAATGGTTCGAGTGAGATTAAAAAGGATGATGGAGA
ACAAATGGAAACAAAAgtgagattttaagaaattattttattaaaagtgatgaaaattcagG
CTCTACCAACATTTCCATTCTCAATTGACAGTATTTTGGCAGTTAGTAGAGTGCCTCGAGGC
AGGCGACCAAATGCGAAATATCCCAGGGTTCAGgtaatttgtttcgtatagtttttcgacaa
attttacaaaactattttcagGCCTGCAAAAACCTATCACCATTTATGATTCCTTTGTTTCC
AATCACTCAGCCTGGTGGGAATGTAATTCGTGAAAAGTCTCCCCCTCTTCCA**AC**GCAACAAT
CTCAGATCGTAGCGACTAATGCATTGACT**AC**CGTTGCTGAATTACTGAAAAGTGTATAAgct
gtctcatcctactttcacctagttaactgcttgtcttaaaatctatgcttctctttagtatc
taaaattttcctagaagcttacaagtatataaatggtctcttctcaataaaggttgtatatt
tattcatcttattgaatctgccatttcctcgtttttgcgagtttatataccttccaattttc
tttctattgtattttcaacttctaattttaattcagggaaactgcttcaacgcatcATGCCA
CAATTCGacATTCTCTGTAAGACACCACCAAAAGTCCTCGTCCGTCAATTCGTCGAGCGTTT
CGAGCGTCCATCCGGAGAGAAGATCGCCCTCTGCGCCGCCGAGCTCACCTACCTCTGCTGGA
TGATCACCCACAACGGAACCGCCATCAAGCGTGCCACCTTCATGTCCTACAACACCATCATC
TCCAACTCCCTCTCCTTCGACATCGTCAACAAGTCCCTCCAATTCAAGTACAAGACCCAAAA
GGCCACCATCCTCGAGGCCTCCCTCAAGgtaagtttaaacatatatatactaactaaccctg
attatttaaattttcagAAGCTCATCCCAGCCTGGGAGTTCACCATCATCCCATACTACGGA
CAAAAGCACCAATCCGACATCACCGACATCGTCTCCTCCCTCCAACTCCAATTCGAGTCCTC
CGAGGAGGCCGACAAGGGAAACTCCCACTCCAAGAAGATGCTCAAGGCCCTCCTCTCCGAGG
GAGAGTCCATCTGGGAGATCACCGAGAAGATCCTCAACTCCTTCGAGTACACCTCCCGTTTC
ACCAAGACCAAGACCCTCTACCAATTCCTCTTCCTCGCCACCTTCATCAACTGCGGACGTTT
CTCCGACATCAAGAACGTCGACCCAAAGTCCTTCAAGCTCGTCCAAAACAAGTACCTCGGAG
TCATCATCCAATGCCTCGTCACCGAGACCAAGACCTCCGTCTCCCGTCACATCTACTTCTTC
TCCGCCCGTGGACGTATCGACCCACTCGTCTACCTCGACGAGTTCCTCCGTAACTCCGAGCC
AGTCCTCAAGCGTGTCAACCGTACCGGAAACTCCTCCTCCAACAAGCAAGAGTACCAACTCC
TCAAGGACAACCTCGTCCGTTCCTACAACAAGGCCCTCAAGAAGAACGCCCCATACTCCATC
TTCGCCATCAAGAACGGACCAAAGTCCCACATCGGACGTCACCTCATGACCTCCTTCCTCTC
CATGAAGgtaagtttaaacatgattttactaactaactaatctgatttaaattttcagGGAC
TCACCGAGCTCACCAACGTCGTCGGAAACTGGTCCGACAAGCGTGCCTCCGCCGTCGCCCGT
ACCACCTACACCCACCAAATCACCGCCATCCCAGACCACTACTTCGCCCTCGTCTCCCGTTA
CTACGCCTACGACCCAATCTCCAAGGAGATGATCGCCCTCAAGGACGAGACCAACCCAATCG
AGGAGTGGCAACACATCGAGCAACTCAAGGGATCCGCCGAGGGATCCATCCGTTACCCAGCC
TGGAACGGAATCATCTCCCAAGAGGTCCTCGACTACCTCTCCTCCTACATCAACCGTCGTAT
CTAAatttttttaaaattcaattttgaaccgtgccctccattggttatttgtacctccagtt
tctcttcttcatttctaagattcaaatttaataaattttatcaaaatgatcaggattgctat
ttttgttcttcttatgctatcattggtaagttataatgcgagctctctacgtaggcatcaca
atgcgtgcctacggcaatctcgacctattcttctttttccgcgaagaatattattactagac
acaaaaagtctgtattgaaggcatgtaggcagacatttttttgccttcttgggaaagaatttt
atagaggaattcagcaatatcaagttttcaagtatatcattgtcaggtagttctcgttgaat
cgaataatttcaatcatctggatcaacgaactcgatgtgagacgaagttcgactgttcctgg
taccaattttgtccggctggtcggtgcttgaaca

PHX7929

>srx-9(syb7929[srx-9::SL2::FLP D5])

catcaagtggggaaataagtttacaaaagggaataacatttaaacggcaagcaaaaactga
tacgtttagtttatctcagcttagacaaccggttccaaacggcaagattggcaagtgctgga
ttaactttttcagGTACCTTCTCCAATTTATGTTCACTTCAGTTTCTATATCACCACTTG
GGTTATATTTGAAATACTGCAGTATATAATTCCTGACAAAAAGTTTCAACCACACGCAATCA
TACCAGCAATGATTATACTTAACTGCTCATCAAATTCTGTAATTTTTCTTTCTATTAACAAG
GAAGTAAAATGCTCGTTCCAATGTTCATGGTTTATCGACAAACTGGGATGTAAG**AAAGCT**AC
TGGAAACAGTCAAGTTACTCCGGTCAATTTAATTTGAgctgtctcatcctactttcacctag
ttaactgcttgtcttaaaatctatgcttctctttagtatctaaaattttcctagaagcttac
aagtatataaatggtctcttctcaataaaggttgtatatttattcatcttattgaatctgcc
atttcctcgtttttgcgagtttatataccttccaattttctttctattgtattttcaacttc
taattttaattcagggaaactgcttcaacgcatcATGCCACAATTCGacATTCTCTGTAAGA
CACCACCAAAAGTCCTCGTCCGTCAATTCGTCGAGCGTTTCGAGCGTCCATCCGGAGAGAAG
ATCGCCCTCTGCGCCGCCGAGCTCACCTACCTCTGCTGGATGATCACCCACAACGGAACCGC
CATCAAGCGTGCCACCTTCATGTCCTACAACACCATCATCTCCAACTCCCTCTCCTTCGACA
TCGTCAACAAGTCCCTCCAATTCAAGTACAAGACCCAAAAGGCCACCATCCTCGAGGCCTCC
CTCAAGgtaagtttaaacatatatatactaactaaccctgattatttaaattttcagAAGCT
CATCCCAGCCTGGGAGTTCACCATCATCCCATACTACGGACAAAAGCACCAATCCGACATCA
CCGACATCGTCTCCTCCCTCCAACTCCAATTCGAGTCCTCCGAGGAGGCCGACAAGGGAAAC
TCCCACTCCAAGAAGATGCTCAAGGCCCTCCTCTCCGAGGGAGAGTCCATCTGGGAGATCAC
CGAGAAGATCCTCAACTCCTTCGAGTACACCTCCCGTTTCACCAAGACCAAGACCCTCTACC
AATTCCTCTTCCTCGCCACCTTCATCAACTGCGGACGTTTCTCCGACATCAAGAACGTCGAC
CCAAAGTCCTTCAAGCTCGTCCAAAACAAGTACCTCGGAGTCATCATCCAATGCCTCGTCAC
CGAGACCAAGACCTCCGTCTCCCGTCACATCTACTTCTTCTCCGCCCGTGGACGTATCGACC
CACTCGTCTACCTCGACGAGTTCCTCCGTAACTCCGAGCCAGTCCTCAAGCGTGTCAACCGT
ACCGGAAACTCCTCCTCCAACAAGCAAGAGTACCAACTCCTCAAGGACAACCTCGTCCGTTC
CTACAACAAGGCCCTCAAGAAGAACGCCCCATACTCCATCTTCGCCATCAAGAACGGACCAA
AGTCCCACATCGGACGTCACCTCATGACCTCCTTCCTCTCCATGAAGgtaagtttaaacatg
attttactaactaactaatctgatttaaattttcagGGACTCACCGAGCTCACCAACGTCGT
CGGAAACTGGTCCGACAAGCGTGCCTCCGCCGTCGCCCGTACCACCTACACCCACCAAATCA
CCGCCATCCCAGACCACTACTTCGCCCTCGTCTCCCGTTACTACGCCTACGACCCAATCTCC
AAGGAGATGATCGCCCTCAAGGACGAGACCAACCCAATCGAGGAGTGGCAACACATCGAGCA
ACTCAAGGGATCCGCCGAGGGATCCATCCGTTACCCAGCCTGGAACGGAATCATCTCCCAAG
AGGTCCTCGACTACCTCTCCTCCTACATCAACCGTCGTATCTAAataattcaaatctcttta
tattaatgatactaataatagtttcttttaataaatacacaaacatcgctttacgtatttca
agggccgggaactttacagtgtggcaacaatcaaccacgtccaaggctttgtttttcccgc
cctttttttttatttctttgcccttttttttggatggaaaaaaatttggccgtggtggag
agtttaactgtgactaaattgtttgtttgtttttccggccctttttttttcttttcccttt
ttttttggatggaaaaaaatgtgtccgtggtggagagttgaactgtgactaaattgtttgtt
tgtttaattgatgattttcccactgatttgtctacaaagggcatcgaaaagcacccaatatt
tagagaacagaaaattttgagaattactgccttcagaaattgatgattttcccactgatttg
tctacaaagggcatcgaaaagcacccaatatttagagaacagaagattttgagaattactgc
cttcagaaattgacaattttcccattgatttgtctacaaagggcattaaaagcatccaatat

ttagagttggtacatgaatgtgcgggtgt

PHX6265

>syb2346syb6265[flp-11p::FLP D5::flp-11 3’UTR]) III:7007600.

cagtaaagatcaaaacgtagccgttgggaagcgtatagatatactcgacactgtaacttgaa
aaactgcagaagttaaaaaatgaatttgcacaggctgcgctagcctctccagttttattggt
atttcaaatcattttctcgtctacttggtgcaacacgcgtgcctctgtagcaaagactgaac
tacttcgcatacttccatgtatttaacacgtcgtcttgtaaaaatagtttgagagatttttt
caccattctcgttttccgtattcattcaacaaccgatgaaaaatccgtatttttcccgaata
aatattcagtgattgcgagaagaaattattttaatgatcaaactctaatgattcttgataaa
gaatgtattgttttgtaaaataaagaaaacaagtattctaggtaacatattaacctgggaac
aataagtcggtgaagtgatgtcattgaaaatacataatggcgatgtcattttcatgttatta
tagttaaagactgtgattttttaattataaattgtaattaattattgatgttctagttaagtt
tggttgattgaagaaattgtatttttttaacttttgtaagggatatgactttttttgagttaaa
taaatgaggtatatagttaaataacacaccagatgatgtgagtgggcctgggtcgagctgca
cattttggagaaacccaaaaaccgtgtttcataagtttgaatacaatgactttgtttttttg
aaggattttgtgcagcgttgttttaaaaaacggttttcataagatttttatcacagttgaa
ctcctatatttcttccctcactaattcttgatgtgtggacaaaactgtaagtctgatcaagg
caacaacacaacatagaccacttttctagtccagtaaaaaatcgaactctgatattcagact
ggtatcacttcttagagtcagaagatggtacagcttgggaaaaacttggaaaaactatccca
gactgccaaaactgaagcttaaagtgagcacaaaattgctccaaactttttttgcagctcac
attcggtccaagtgttgttcaatttatgatgattatcagtctgttttgccaaattcggatcc
aactttttttttttttttaggagaatttattttaaagctcgccattgaaatacatgtaaaaa
tctaagcttttaaatttaacagaaaatgagattatctttttttggaatgaaacctagaatttt
caaaataactagcatctcaaaataactagcatccaagatgctctttgttttcataaattta
gttgtttccagtcccgaaaaaaaatttcaatcatttccttaggttcaataagtgttttgtct
ggtctcttgttgcaattttttgaaaaaggaataacattgtgctcagactgaaaaatgaaaaaa
gatgatgtaagttggtctaggctttaattccagtttccgttccactagtgatcttatcattt
attcatatcaagtttcatttgatgttcgattcattgtaggtttcattgttccctctgactat
tcatcaagttctatgaaaccattgtggcttactagttccccaaagttggaaagatctggaga
aagaaaaacaagtatcccaaaagcccaaaccttttttttccaaatgttgagcctctaaagtt
gatgaatggggtgagagaggttttcatggttttatggcccatcagttccagtagagttcctc
atatcttttcgcacttcatttttcaatctgttaagagtaattcccccacaacttttagcatc
ctcaatcctggttccacccacaaacacccacactccattgaatataagattataaaatataa
gttaaaatacgaaaagatgcaaaactgtgacgacgaaacttaacaacctgctgacgtgtcaa
atatttccgcatcttaccttcgtgggcatcttctaactcataaatttatgatgttcgaatcc
cggcgccaagtgatgtaaactcatttaatatttgaggaaaaagcatagacgaatccattttg
catctaatttatttttctagtttcacacccatgtttcgcttggcataatcagtgtttgagcg
cactttttgcaaggtgcgtcttcttgtaaagaaagattttcaaaaaagtgcaaaagtgggg
agattgaaaaacgataagaaggtttcatgtcaaacaaggacacacgaatatataaatacggg
tcatttttcaaactttccattgtgactttttggaatattttttttcgtaaaaatgaaatcgat
tttctgaccaatatctcaactttaaaacttctaattctgctttttttcaatcttctgtaaac
aggagtgcgtcgaaatttcggcagtttcggcatattgccaaaagtttatagtccaacaattg
ccggaactggtgatttagctaaacaataaacctcaaaatattaggtatttcagtaagaaaat
gacctattgattatgactttgaatcaagttttgatatatttttaatttttgaaatatgatga
aaattaaaaattatcaaaaattctggcaattttggcacatcggcacacaatgcgaaatttgc

ggcaactgccgattttaaaaactttatttgtttggtataacagttaggcacgcattttgg
aaataaaccgatcttttattgggagaagtgccagttaaagtgctggaccaagtaaaatgttt
ccaaaaaaccagacacgtattaataaaacacttcaaactttttcaaattttacaacaatttt
caattaaagttttggcaaattggtaaattttcaagaaatttttttgcaagttttaattttta
aatatacattataattattagaatgttttctgtatagaaaatctctgcatctgttggaaatg
aataaaaacttcataacaattctaaattaaaaattcaagaacactagaacaagcgtcctcaa
aatattcacaaaatattgtacacattttgtagtactttttccattattaaaaaaagcaaaac
tagcttttccttcctttccgaaatttaatgctattttcaagatgactttttttgcttgcgttt
ttctcagtttcctcacacacacacacacacaagtaggcgtggcctgtggaacgtttcagagc
gcagaacacctgcatttgatctattcacttcttgcttttgaaaagcccaaagacaccctaca
cttcggtttcgttttggaaaccattgacatcatcctattttccataagaagtttccttgaga
agaatccatttcgcaaatttttcattaaaacgttcaaaactcatcaaaccatttgtaaatag
taataaagtatgtcctgcggctatttgctttctcttcggaatctacaacgccccctcctaat
acatcgtttcaggtataaaaagactgcgcctagccgctcgtctcacttttttgcagttcatac
tgaata*aaaaa*ATGCCACAATTCGacATTCTCTGTAAGACACCACCAAAAGTCCTCGTCCGT
CAATTCGTCGAGCGTTTCGAGCGTCCATCCGGAGAGAAGATCGCCCTCTGCGCCGCCGAGCT
CACCTACCTCTGCTGGATGATCACCCACAACGGAACCGCCATCAAGCGTGCCACCTTCATGT
CCTACAACACCATCATCTCCAACTCCCTCTCCTTCGACATCGTCAACAAGTCCCTCCAATTC
AAGTACAAGACCCAAAAGGCCACCATCCTCGAGGCCTCCCTCAAGgtaagtttaaacatata
tatactaactaaccctgattatttaaattttcagAAGCTCATCCCAGCCTGGGAGTTCACCA
TCATCCCATACTACGGACAAAAGCACCAATCCGACATCACCGACATCGTCTCCTCCCTCCAA
CTCCAATTCGAGTCCTCCGAGGAGGCCGACAAGGGAAACTCCCACTCCAAGAAGATGCTCAA
GGCCCTCCTCTCCGAGGGAGAGTCCATCTGGGAGATCACCGAGAAGATCCTCAACTCCTTCG
AGTACACCTCCCGTTTCACCAAGACCAAGACCCTCTACCAATTCCTCTTCCTCGCCACCTTC
ATCAACTGCGGACGTTTCTCCGACATCAAGAACGTCGACCCAAAGTCCTTCAAGCTCGTCCA
AAACAAGTACCTCGGAGTCATCATCCAATGCCTCGTCACCGAGACCAAGACCTCCGTCTCCC
GTCACATCTACTTCTTCTCCGCCCGTGGACGTATCGACCCACTCGTCTACCTCGACGAGTTC
CTCCGTAACTCCGAGCCAGTCCTCAAGCGTGTCAACCGTACCGGAAACTCCTCCTCCAACAA
GCAAGAGTACCAACTCCTCAAGGACAACCTCGTCCGTTCCTACAACAAGGCCCTCAAGAAGA
ACGCCCCATACTCCATCTTCGCCATCAAGAACGGACCAAAGTCCCACATCGGACGTCACCTC
ATGACCTCCTTCCTCTCCATGAAGgtaagtttaaacatgattttactaactaactaatctga
tttaaattttcagGGACTCACCGAGCTCACCAACGTCGTCGGAAACTGGTCCGACAAGCGTG
CCTCCGCCGTCGCCCGTACCACCTACACCCACCAAATCACCGCCATCCCAGACCACTACTTC
GCCCTCGTCTCCCGTTACTACGCCTACGACCCAATCTCCAAGGAGATGATCGCCCTCAAGGA
CGAGACCAACCCAATCGAGGAGTGGCAACACATCGAGCAACTCAAGGGATCCGCCGAGGGAT
CCATCCGTTACCCAGCCTGGAACGGAATCATCTCCCAAGAGGTCCTCGACTACCTCTCCTCC
TACATCAACCGTCGTATCTAAaaatcatatgtttttctctctcacactctctttttttcatac
tctctcttgctgtctagaatttgattggtgtcgcttaaccccccctttccctccgaaggaaag
ttatctccccagatctcttttggtgtttttttatcagctaacaacacacattttctgatattt
ctatgctctgtctatgaacaataaaggcgttgttaattactcgcaaaatcactttgtttatt
tttttcacattttcagatagtgaacaaaagaaaattaaattctaaaatctgaatcggaaaat
tcaaattaaaaattaaatttattttttttatattacacctgttttttttcaaatattagatc
aaaaactattcaacaagtggcatgtaaagcataacgaggtatatgggcttcagatcttcaac
tggagagcatcattaaaatcagataaccgtcaaacaactgggatagaagtgtttccttctga

aagtttaccaacttttcaaaaattaatccgaaattattctaccatccaattcgtttgatctt
gattttgattggcaaattgtaaaataaattataaaattatgatttctcccactttggaccgc
cagacattttttatatttccacccactttttcttgaaacgtcatatcgtgttagtcgaacat
ccattttctttcttgaaagatatatcatgaaattcagtgatagaagcaaaaaaaaaacgaaa
aaaaaggaacatgcatttcagaagacacttccttatgaggatcatttataacagaataactt
tggccacaaatatatgaatttgcagccgctttctcggaaattgccaa

PHX8127

>syb8127[dpy-10site::unc-25p fragment with tataa sites::dpy-10site::sl1-aaaa::FLP D5::let-858 3'utr]) IV:5015000.

GGTTCAAGAAGTCACTCGACTATCATTAATGTTTTCAATAGATAGcaatattgtttcacatt
ttgctgtgaagttttgtgaaattttgccccaaaacaagttggaggcaattttgaaatgaac
aaattttaatcatatttggagaaaatagcaggaaaataagaaatttggccgtgtgtctttaa
aaagtatagtagtttccacgcaagtacacgattgagctgatgggtaagaaaagaactttgca
gtttggttgtagtgaacaataaattcgtttttttatttggtttacttttttcttggtttttgt
atttttacacgggccctactgtactcactacagtagtcacacagttcatcgactagtgtgag
acaagtgtgaaactaaagtaccgctcgtggtgcctatggtagcggagctataattgttttct
atttcatggccggtaaattattttttttctttctttttttttgctcttttttttcaagaatt
ttcgaattgtttgaagggctgctcatctataattttgtcattttgttctgatgccatcattt
ctgagaggacctttgaagactcgtcacgaaacgggagggggggctcaagtgagcattattatt
attattattgtcgcaaaaagtttaccccgggcgctccgctaccataggcaccacgagcggta
ctataaaagtttaaacatatatatactaactaaccctgattatttaaattttcaggaaaaAT
GCCACAATTCGACATTCTCTGTAAGACACCACCAAAAGTCCTCGTCCGTCAATTCGTCGAGC
GTTTCGAGCGTCCATCCGGAGAGAAGATCGCCCTCTGCGCCGCCGAGCTCACCTACCTCTGC
TGGATGATCACCCACAACGGAACCGCCATCAAGCGTGCCACCTTCATGTCCTACAACACCAT
CATCTCCAACTCCCTCTCCTTCGACATCGTCAACAAGTCCCTCCAATTCAAGTACAAGACCC
AAAAGGCCACCATCCTCGAGGCCTCCCTCAAGgtaagtttaaacatatatatactaactaac
cctgattatttaaattttcagAAGCTCATCCCAGCCTGGGAGTTCACCATCATCCCATACTA
CGGACAAAAGCACCAATCCGACATCACCGACATCGTCTCCTCCCTCCAACTCCAATTCGAGT
CCTCCGAGGAGGCCGACAAGGGAAACTCCCACTCCAAGAAGATGCTCAAGGCCCTCCTCTCC
GAGGGAGAGTCCATCTGGGAGATCACCGAGAAGATCCTCAACTCCTTCGAGTACACCTCCCG
TTTCACCAAGACCAAGACCCTCTACCAATTCCTCTTCCTCGCCACCTTCATCAACTGCGGAC
GTTTCTCCGACATCAAGAACGTCGACCCAAAGTCCTTCAAGCTCGTCCAAAACAAGTACCTC
GGAGTCATCATCCAATGCCTCGTCACCGAGACCAAGACCTCCGTCTCCCGTCACATCTACTT
CTTCTCCGCCCGTGGACGTATCGACCCACTCGTCTACCTCGACGAGTTCCTCCGTAACTCCG
AGCCAGTCCTCAAGCGTGTCAACCGTACCGGAAACTCCTCCTCCAACAAGCAAGAGTACCAA
CTCCTCAAGGACAACCTCGTCCGTTCCTACAACAAGGCCCTCAAGAAGAACGCCCCATACTC
CATCTTCGCCATCAAGAACGGACCAAAGTCCCACATCGGACGTCACCTCATGACCTCCTTCC
TCTCCATGAAGgtaagtttaaacatgattttactaactaactaatctgatttaaattttcag
GGACTCACCGAGCTCACCAACGTCGTCGGAAACTGGTCCGACAAGCGTGCCTCCGCCGTCGC
CCGTACCACCTACACCCACCAAATCACCGCCATCCCAGACCACTACTTCGCCCTCGTCTCCC
GTTACTACGCCTACGACCCAATCTCCAAGGAGATGATCGCCCTCAAGGACGAGACCAACCCA
ATCGAGGAGTGGCAACACATCGAGCAACTCAAGGGATCCGCCGAGGGATCCATCCGTTACCC
AGCCTGGAACGGAATCATCTCCCAAGAGGTCCTCGACTACCTCTCCTCCTACATCAACCGTC
GTATCTAAattttcaaattttaaatactgaatatttgtttttttcctattatttatttatt
ctctttgtgtttttttcttgctttctaaaaaattaattcaatccaaatctaaacatttttt
tttctctttccgtctcccaattcgtattccgctcctctcatctgaacacaatgtgcaagttt
atttatcttctcgctttcatttcattaggacgtgggggaattggtggaaggggaaacaca
caaaaggatgatggaaatgaaataaggacacacaatatgcaacaacattcaattcagaaata
tggaggaaggtttaaaagaaaacataaaaatatatagaggaggaaggaaaactagtaaaaaa
taagcaaagaaattaggcgaacgatgagaattgtcctcgcttggcttaggcgacattttgtt

aataatcgtaacttaaaggaggactaacagttcgggcgattttgatcttatagacccaacat
gggctcaaatgaacggattgaaacttctcgaaaacttttcaaactttttcatgccggttcaa
aattttgaaaaagctacactgattttagtcaaaatctcgaattttggccatttttccgtctc
acatctgtcggaagacaaaactgttaacaaaactgttaatacccaactttttttaactaaaaa
aaattaatatggaaacgttggaaacagttcattcgatctgaaaagtataaagctaaaaaaat
aagtgaatgtagaaaatctttattttcaaagaggatcctttctttatttacgcaacgcca
