# Supplementary material for: The neuropeptide FLP-11 induces and self-inhibits sleep through the receptor DMSR-1 in Caenorhabditis elegans
Source: Curr Biol. Author manuscript; Available in PMC 2025 Jun 24. (PMC7617803; doi:10.1016/j.cub.2025.03.039)
Supplement: Document S1. Figures S1–S4. [file EMS206526-supplement-Document_S1__Figures_S1_S4_.pdf]

Current Biology, Volume *35*

Supplemental Information

The neuropeptide FLP-11 induces and self-inhibits sleep through the receptor DMSR-1 in *Caenorhabditis elegans*

Lorenzo Rossi, Kenneth Amoako, Inka Busack, Luca Golinelli, Amy Courtney, Judith Besseling, William Schafer, Isabel Beets, and Henrik Bringmann

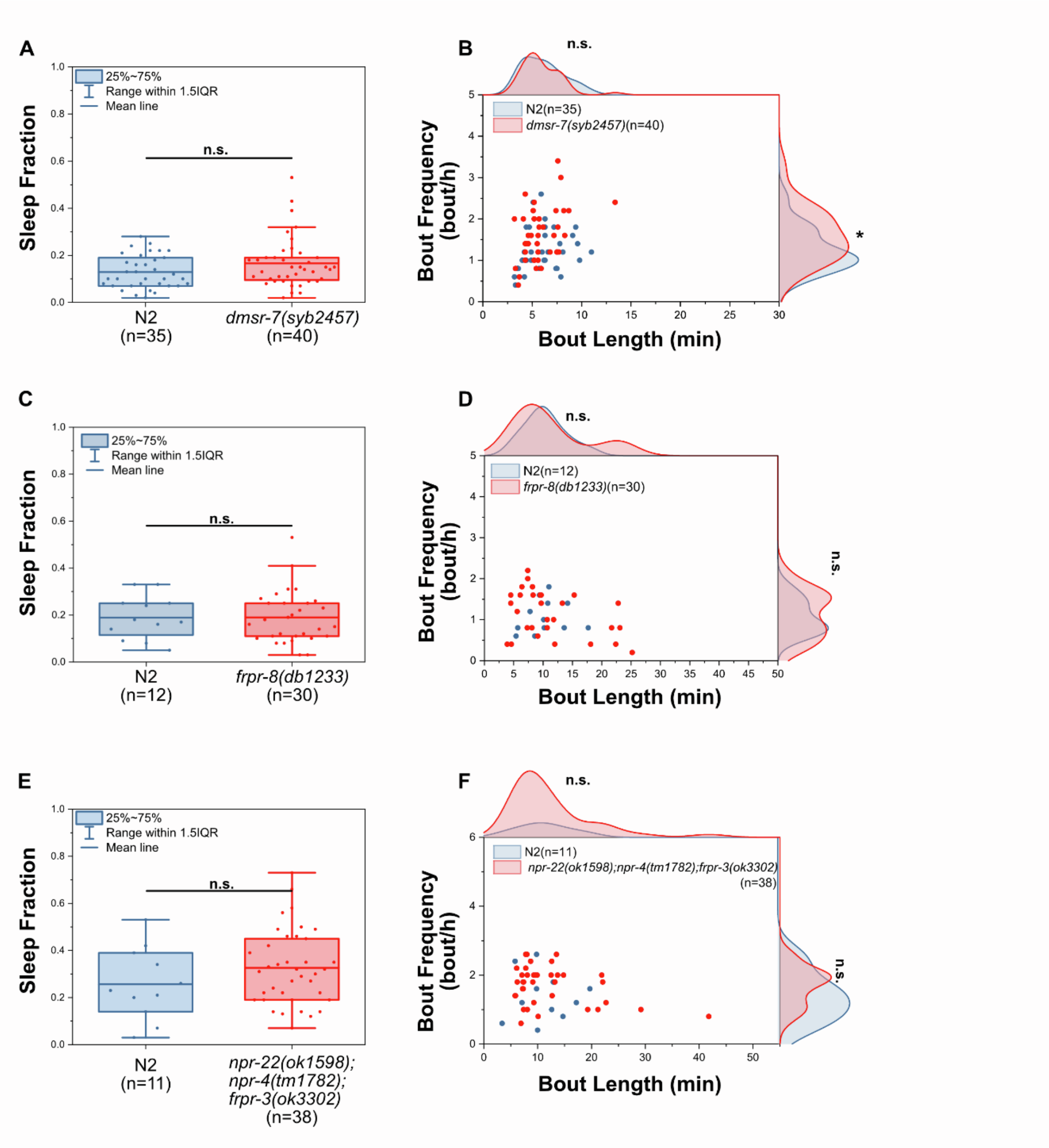

Figure S1. Mutations in FLP-11 receptors other than DMSR-1 do not show any obvious sleep phenotypes. Related to Figure 1

(A-B) *dmsr-7* deletion does not change (A) the fraction of time spent sleeping. (B) Sleep bout length is not significantly changed, and sleep bout frequency is slightly but significantly increased. N2: n = 35, *dmsr-7(syb2457):* n = 40, 3 biological replicates. n.s. = not significant (p > 0.05), * = p ≤ 0.05, Wilcoxon rank-sum test.

(C-D) *frpr-8* deletion does not cause any obvious sleep phenotype. N2: n = 12, *frpr-8(db1233)*: n = 30, 2 biological replicates. (C) The fraction of time spent sleeping, as well as (D) sleep bout

alignment and frequency, is not significantly changed. n.s. = not significant ($p > 0.05$), Wilcoxon rank-sum test.

(E-F) Since *npr-22*, *npr-4*, and *frpr-3* had previously only been tested for sleep in developing larvae, we also included the triple receptor mutant in this screen. The triple mutant does not show significant changes in (E) the fraction of time spent sleeping or (F) sleep bout length and frequency. N2: n = 11, *npr-22(ok1598); npr-4(tm1872); frpr-3(ok3302)*: n = 38, 3 biological replicates. n.s. = not significant ($p > 0.05$), Wilcoxon rank-sum test.

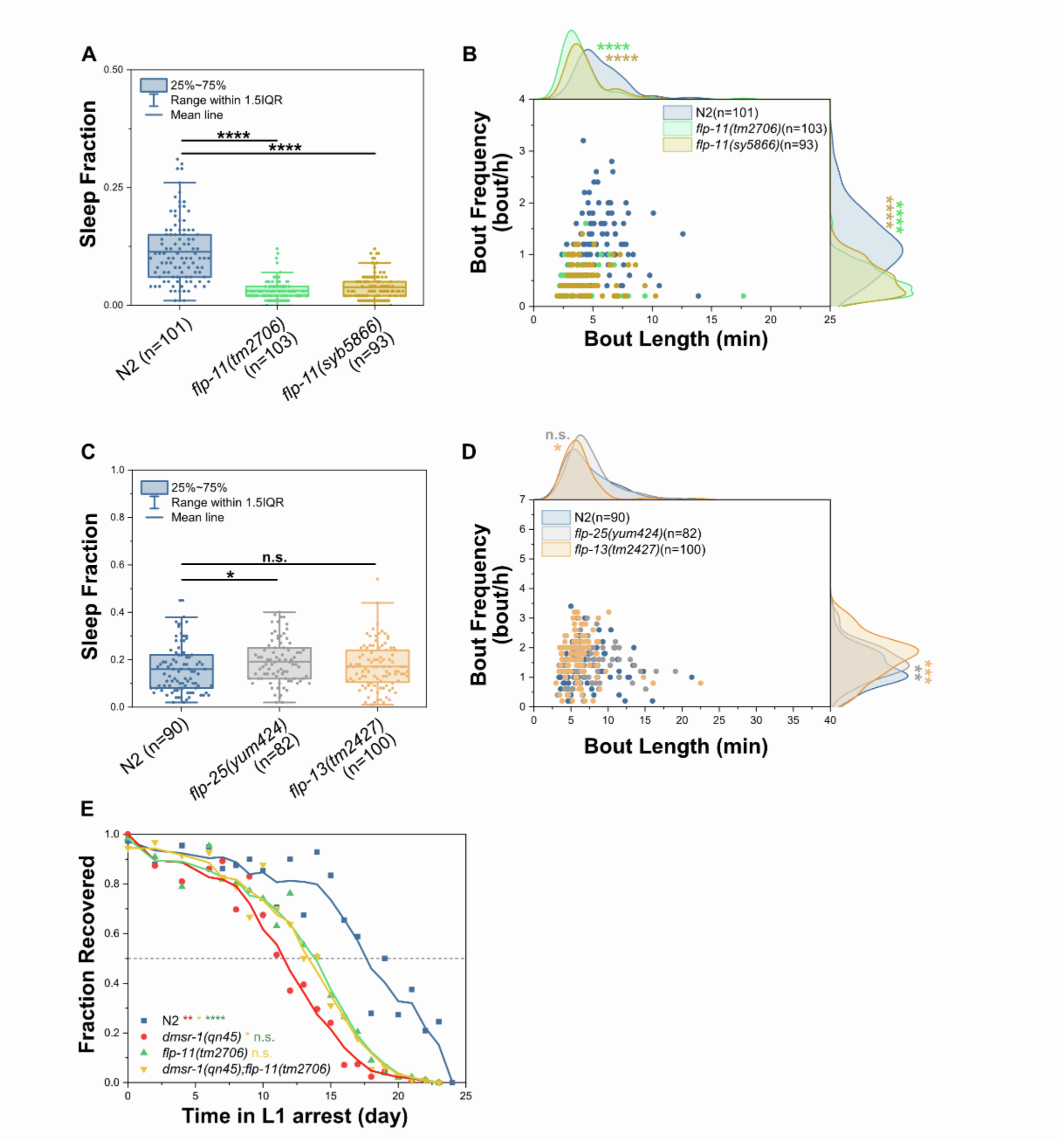

Figure S2. L1 arrest sleep depends on FLP-11, while other DMSR-1 binding peptides are not necessary to sleep. DMSR-1 is also required for recovery, acting on the same molecular pathway as FLP-11. Related to Figure 2

(A-B) The deletion of the coding sequence for the four FLP-11 peptides of the *flp-11(tm2706)* allele, as well as the complete deletion of the coding sequence in the *flp-11(syb5866)* allele both result in a similar sleep phenotype. N2: n = 101, *flp-11(tm2706)*: n = 103, *flp-11(syb5866)*: n = 93, 3 biological replicates. (A) The fraction of time spent sleeping is significantly reduced in both genotypes. (B) The sleep loss is caused by significantly less frequent and shorter sleep bouts. **** = p ≤ 0.0001, Wilcoxon rank-sum test.

(C-D) Sleep in *flp-25* and *flp-13* deletion mutants. (C) Deletion of *flp-25* causes a small increase in the time spent sleeping, while deletion of *flp-13* has no detectable effect on overall sleep duration. (D) The sleep phenotype of *flp-25* mutant larvae is due to an increase in sleep bout frequency. In *flp-13* mutant larvae, the bouts are shorter but occur more frequently; thus, the effects compensate for each other, resulting in no net change in sleep duration. N2: n = 90, *flp-25(yum424)*: n = 82, *flp-13(tm2427)*: n = 100, 3 biological replicates. n.s. = not significant ($p > 0.05$), * = $p \leq 0.05$, ** = $p \leq 0.01$, *** = $p \leq 0.001$, Wilcoxon rank-sum test.

(E) *dmsr-1* is required for recovery from L1 arrest. The *dmsr-1(qn45)*, *flp-11(tm2706)*, and *dmsr-1(qn45); flp-11(tm2706)* double mutants reduce the recovery rate to a similar extent, indicating that they are acting in the same molecular pathway. We averaged survival data from three biological replicates. Approximately 100 animals were assayed per replicate for each genotype at every time point. A Fisher's exact test was conducted at the median recovery of *dmsr-1(qn45)* on day 11, of *flp-11(tm2706)* on day 14, and of *dmsr-1(qn45); flp-11(tm2706)* on day 13. n.s. = not significant ($p > 0.05$); * = $p \leq 0.05$, ** = $p \leq 0.01$, **** = $p \leq 0.0001$.

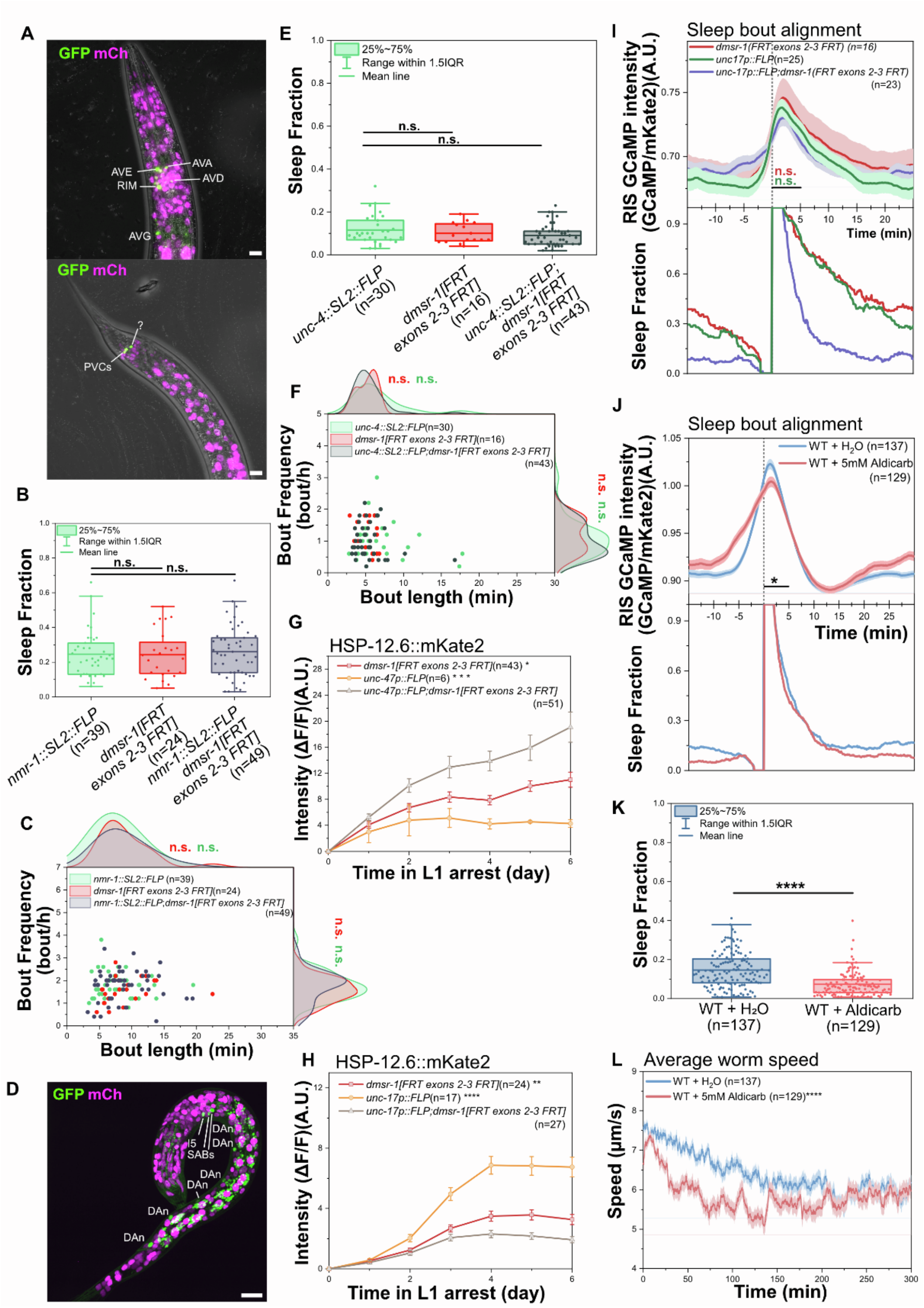

Figure S3. Characterization of conditional *dmsr-1* knockdowns and the effect of aldicarb on movement speed. Related to Figure 3

(A) Characterization of the FLP recombinase knocked into the *nmr-1* locus was performed using a fluorescent protein expression readout. In this reporter system, a heat shock drives the expression of a fluorescent protein under the control of a heat-shock promoter. In the absence of recombination, the mCherry reporter is expressed, whereas, after recombination, GFP is expressed[14]. L4 worms were heat-shocked for 2 hours at 32°C, and imaged 2 hours after the heat shock. *nmr-1::SL2::FLP* causes recombination in command interneurons: AVA, AVD, AVE, RIM, AVG in the head region (upper panel) and PVCs neurons in the tail (lower panel). There was also additional neuron that remains to be identified). We visually inspected at least 10 worms, which showed consistent expression. We imaged two worms for documentation. mCh: mCherry. Scale bar: 10µm.

(B-C) The knockdown of *dmsr-1* in *nmr-1*-expressing neurons did not significantly change the fraction of time spent sleeping, sleep bout length, or frequency. *nmr-1::SL2::FLP*: n = 39, *dmsr-1[FRT exons 2-3 FRT]*: n = 24, *nmr-1::SL2::FLP; dmsr-1[FRT exons 2-3 FRT]*: n = 49, 2 biological replicates. n.s. = not significant ($p > 0.05$), Wilcoxon rank-sum test.

(D) Characterization of the FLP recombinase knocked into the *unc-4* locus was performed using the fluorescent protein expression readout. 48-hour L1 arrested worms were heat-shocked for 2 hours at 32°C, and imaged 2 hours after the heat shock. *unc-4::SL2::FLP* causes recombination specifically in Dorsal A-type motor neurons, SABs neurons and I5. We visually inspected at least 10 worms, which all showed a consistent pattern of recombination and imaged 6 worms for documentation. Recombination occurred in all of the imaged worms. mCh: mCherry. Scale bar: 10µm.

(E-F) The knockdown of *dmsr-1* in *unc-4*-expressing neurons did not significantly change the fraction of time spent sleeping, sleep bout length, or frequency. *unc-4::SL2::FLP*: n = 30, *dmsr-1[FRT exons 2-3 FRT]*: n = 16, *nmr-1::SL2::FLP; dmsr-1[FRT exons 2-3 FRT]*: n = 43, 2 biological replicates. n.s. = not significant ($p > 0.05$), Wilcoxon rank-sum test.

(G) *dmsr-1* knockdown in GABAergic neurons increases the expression of *hsp-12.6*. *dmsr-1[FRT exons 2-3]*: n = 43, *unc-47p::FLP*: n = 6; *dmsr-1[FRT exons 2-3]; unc-47p::FLP*, n = 51, 2 biological

replicates. * = p ≤ 0.05, **** = p ≤ 0.0001, The Wilcoxon rank-sum test was performed after averaging the fluorescence intensity per worm over the last 4 days.

(H) *dmsr-1* knockdown in cholinergic neurons decreases the expression of *hsp-12.6*. *dmsr-1[FRT exons 2-3]*: n = 24, *unc-47p::FLP*: n = 17; *dmsr-1[FRT exons 2-3]; unc-47p::FLP*, n = 27, 2 biological replicates. ** = p ≤ 0.01, **** = p ≤ 0.0001, The Wilcoxon rank-sum test was performed after averaging the fluorescence intensity per worm over the last 4 days.

(I) Sleep bout alignment suggests that knockdown of *dmsr-1* in cholinergic neurons does not significantly alter RIS activity during sleep. *unc-17p::FLP*: n = 25, *dmsr-1[FRT exons 2-3 FRT]*: n = 16, *unc-17p::FLP; dmsr-1[FRT exons 2-3 FRT]*: n = 23, with 3 biological replicates. n.s. = not significant (p > 0.05), Wilcoxon rank-sum test for GCaMP intensity from 0 to 5 minutes.

(J-L) We treated worms with aldicarb and monitored their behavior and RIS calcium activity for 5 hours. Movement speed and sleep were then quantified. The RIS calcium data are presented in Figure 4C. Wild-type background + vehicle (H_2O): n = 137, wild-type background + 5 mM aldicarb (dissolved in H_2O): n = 129, with 2 biological replicates for each condition. (J) Sleep bout alignment suggests lower activation of RIS during sleep. * = p ≤ 0.05, Wilcoxon rank-sum test for GCaMP intensity from 0 to 5 minutes. (K) Worms treated with aldicarb spent less time sleeping compared to control worms. **** = p ≤ 0.0001, Wilcoxon rank-sum test. (L) The average speed of worms treated with aldicarb is significantly different than that of control worms. However, despite an initial decrease in speed, worms never became immobilized in the presence of aldicarb. **** = p ≤ 0.0001, Wilcoxon rank-sum test calculated between the average speed (over 5 hours) of each worm.

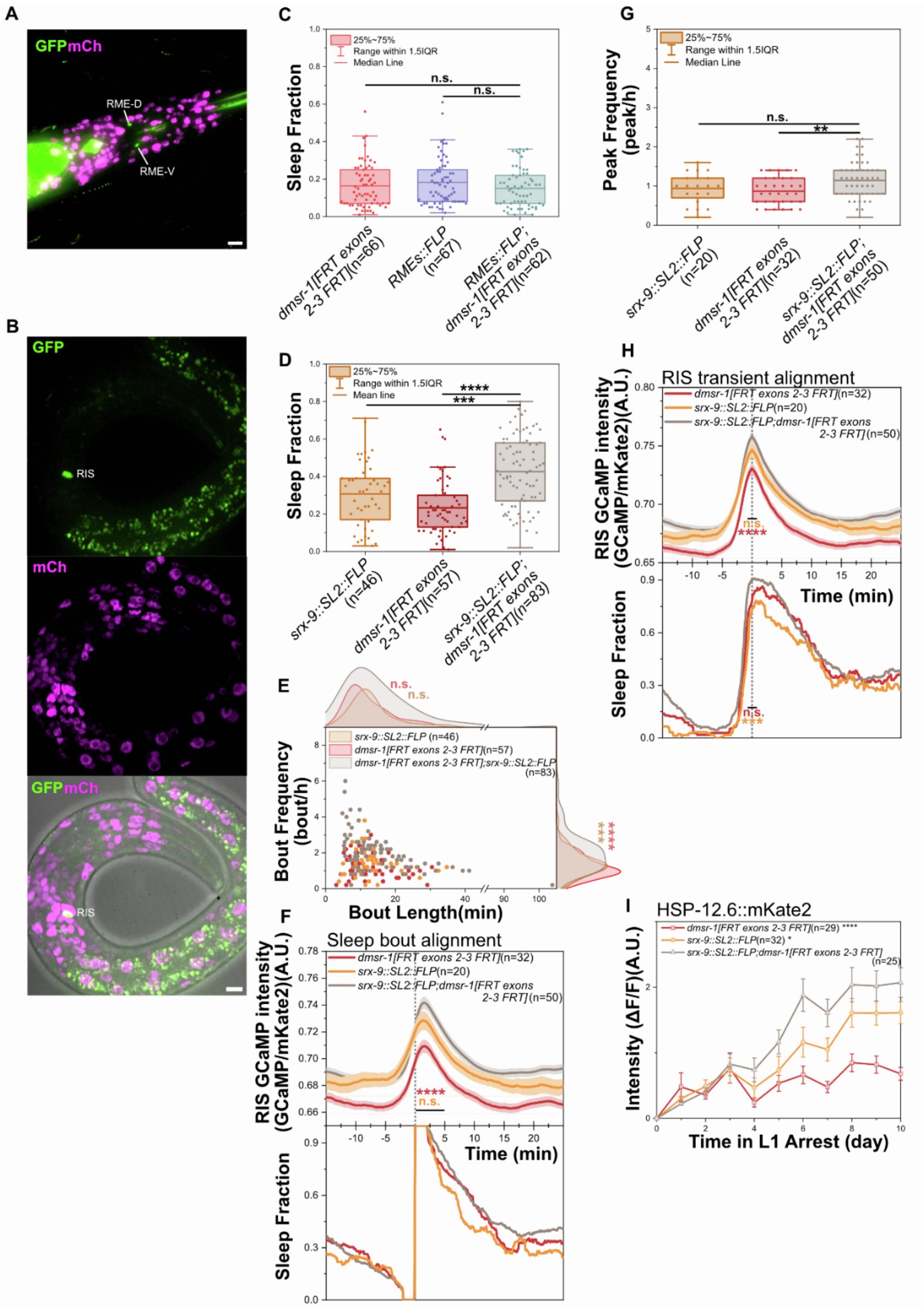

Figure S4. *dmsr-1* acts in RIS to inhibit its activity, thus limiting sleep. Related to Figure 4

(A) Characterization of the RME-specific FLP recombinase strain was performed using the fluorescent protein expression readout. L4 worms were heat-shocked for 2 hours at 32°C, and

imaged 2 hours after the heat shock. We visually inspected at least 10 worms and imaged 7 worms for quantification. Of these imaged worms 1 showed recombination only on the dorsal and ventral RMEs (figure), one in just one of the RME neurons, and one in one of the RMEs and other head neurons. mCh: mCherry. Scale bar: 10 µm.

(B) Characterization of the FLP recombinase knocked into the *srx-9* locus was performed using the fluorescent protein expression readout. 48-hour L1 arrested worms were heat-shocked for 4 hours at 32°C, and imaged 3 hours after the heat shock. *srx-9::SL2::FLP* causes recombination specifically in RIS. Number of worms imaged: n = 25. Recombination occurred in 84% of imaged worms. mCh: mCherry. Scale bar: 10 µm.

(C) *dmsr-1* knockdown in RME neurons does not result in any obvious sleep phenotype. *dmsr-1[FRT exons 2-3 FRT]*: n = 66, *RME::FLP*: n = 67, *RME::FLP*; *dmsr-1[FRT exons 2-3 FRT]*: n = 62, 3 biological replicates. n.s. = not significant ($p > 0.05$), Wilcoxon rank-sum test.

(D-E) *dmsr-1* acts in RIS to inhibit sleep. (F) Knockdown of the receptor specifically in RIS, driven by *srx-9::SL2::FLP*, increases the fraction of time spent sleeping. (G) This increased sleep is primarily due to a significant rise in the frequency of sleep bouts. While both *srx-9::SL2::FLP* and *flp-11p::FLP* drivers cause an increase in sleep, the increased sleep caused by the *flp-11p::FLP* driver is due to longer sleep bouts. This could be due to the *srx-9::SL2::FLP* driver being weaker than the *flp-11p::FLP* driver, or the *flp-11p::FLP* driver being less specific. Since both the *unc-47p::FLP* and *flp-11p::FLP* drivers cause extended sleep bouts, it seems more likely that the primary effect of *dmsr-1* in RIS is to reduce the length of sleep bouts. *dmsr-1[FRT exons 2-3]*: n = 57, *srx-9::SL2::FLP*: n = 46; *dmsr-1[FRT exons 2-3]; srx-9::SL2::FLP*, n = 83, 3 biological replicates. n.s. = not significant ($p > 0.05$), *** = $p \leq 0.001$, **** = $p \leq 0.0001$, Wilcoxon rank-sum test.

(F-H) *dmsr-1* knockdown in RIS using the *srx-9::SL2::FLP* recombinase results in similar, but much weaker, phenotypes compared to the *flp-11p::FLP* recombinase. This difference may be explained by the much weaker expression driven by the *srx-9* promoter. *dmsr-1[FRT exons 2-3 FRT]*: n = 32, *srx-9::SL2::FLP*: n = 20; *dmsr-1[FRT exons 2-3 FRT]; srx-9::SL2::FLP*, n = 50, 3 biological replicates. (F) Sleep bout alignment shows increased RIS activity when comparing the conditional *dmsr-1* knockdown with the conditional *dmsr-1* allele. However, it does not show significantly increased

RIS activity during sleep when comparing the conditional knockdown with the *srx-9::SL2::FLP* recombinase control, possibly because RIS activity is already elevated in the presence of this recombinase driver. n.s. = not significant ($p > 0.05$), **** = $p \leq 0.0001$, Wilcoxon rank-sum test for GCaMP intensity averaged from 0 to 5 minutes. (G) RIS activation peak frequency in the conditional knockdown of *dmsr-1* is increased compared to the conditional *dmsr-1* allele control, but not compared to the *srx-9::SL2::FLP* recombinase control. n.s. = not significant ($p > 0.05$), ** = $p \leq 0.01$, Wilcoxon rank-sum test. (H) RIS peak alignment shows increased RIS activity when comparing the conditional *dmsr-1* knockdown with the conditional *dmsr-1* allele. However, it does not show significantly increased RIS activity during sleep when comparing the conditional knockdown with the *srx-9::SL2::FLP* recombinase control, possibly because RIS activity is already elevated in the presence of this recombinase driver. Sleep during RIS peak activity is elevated when comparing the conditional *dmsr-1* knockdown with the *srx-9::SL2::FLP* recombinase control. However, sleep is not significantly increased when comparing the conditional knockdown with the conditional *dmsr-1* allele, possibly because sleep is already elevated in the presence of the conditional *dmsr-1* allele. n.s. = not significant ($p > 0.05$), *** = $p \leq 0.001$, **** = $p \leq 0.0001$. Wilcoxon rank-sum test for averaged GCaMP intensity and sleep fraction per worm from -1 to +1 minute.

(I) *dmsr-1* knockdown in RIS using *srx-9::SL2::FLP* recombinase increases the expression of *hsp-12.6. dmsr-1[FRT exons 2-3 FRT]*: n = 29, *srx-9::SL2::FLP*: n = 32, *dmsr-1[FRT exons 2-3 FRT]; srx-9::SL2::FLP*, n = 25, 1 biological replicate. This experiment was carried out only in one replicate as it is redundant with Figure 4G. * = $p \leq 0.05$, **** = $p \leq 0.0001$, The Wilcoxon rank-sum test was performed by averaging the fluorescence intensity per worm over the last 4 days.
